# The gap-free genome of mulberry elucidates the architecture and evolution of polycentric chromosomes

**DOI:** 10.1093/hr/uhad111

**Published:** 2023-05-31

**Authors:** Bi Ma, Honghong Wang, Jingchun Liu, Lin Chen, Xiaoyu Xia, Wuqi Wei, Zhen Yang, Jianglian Yuan, Yiwei Luo, Ningjia He

**Affiliations:** State Key Laboratory of Resource Insects, Southwest University, Chongqing, 400715, China; State Key Laboratory of Resource Insects, Southwest University, Chongqing, 400715, China; State Key Laboratory of Resource Insects, Southwest University, Chongqing, 400715, China; State Key Laboratory of Resource Insects, Southwest University, Chongqing, 400715, China; State Key Laboratory of Resource Insects, Southwest University, Chongqing, 400715, China; State Key Laboratory of Resource Insects, Southwest University, Chongqing, 400715, China; State Key Laboratory of Resource Insects, Southwest University, Chongqing, 400715, China; State Key Laboratory of Resource Insects, Southwest University, Chongqing, 400715, China; State Key Laboratory of Resource Insects, Southwest University, Chongqing, 400715, China; State Key Laboratory of Resource Insects, Southwest University, Chongqing, 400715, China

## Abstract

Mulberry is a fundamental component of the global sericulture industry, and its positive impact on our health and the environment cannot be overstated. However, the mulberry reference genomes reported previously remained unassembled or unplaced sequences. Here, we report the assembly and analysis of the telomere-to-telomere gap-free reference genome of the mulberry species, *Morus notabilis*, which has emerged as an important reference in mulberry gene function research and genetic improvement. The mulberry gap-free reference genome produced here provides an unprecedented opportunity for us to study the structure and function of centromeres. Our results revealed that all mulberry centromeric regions share conserved centromeric satellite repeats with different copies. Strikingly, we found that *M. notabilis* is a species with polycentric chromosomes and the only reported polycentric chromosome species up to now. We propose a compelling model that explains the formation mechanism of new centromeres and addresses the unsolved scientific question of the chromosome fusion-fission cycle in mulberry species. Our study sheds light on the functional genomics, chromosome evolution, and genetic improvement of mulberry species.

## Introduction

Mulberry trees not only occupy an important position in the sericulture industry, but also play a crucial role in environmental protection. The first draft genome of mulberry, *Morus notabilis,* was reported in 2013 [1]. Over the past ten years, *M. notabilis* has emerged as an important model system in mulberry gene [2], phenotype [3], genomics [4, 5], and other omics studies [6]. Although several high-quality mulberry genomes, including *M. notabilis* [5, 7], *M. alba* [8], *M. indica* [9], and *M. yunnanensis* [7] have been reported in recent years, there are still numerous unassembled or unplaced sequences.

As the final frontiers of genome projects, centromere regions are the last and most difficult ones to deal with. They contain extremely high contents of repeats, hindering their assembly. In the past two years, the telomere-to-telomere (T2T) or gap-less genome of some plant species, including rice [10–12], *Arabidopsis thaliana* [13–15], banana [16], watermelon [17], barley [18], bitter melon [19], *Brassica rapa* [20], *Rhodomyrtus tomentosa* [21], and strawberry [22] has been reported. Surprisingly, all centromeric regions of those species were successfully assembled. Considering that mulberry has multiple different ploidy levels [23], and centromeres not only play roles in cell division but also in determining large-scale genome architecture and chromatin composition [24], studying complete centromeres can deepen our understanding of the evolutionary process of this species. However, the lack of a mulberry T2T reference genome has prevented detailed studies on its intriguing centromere organization and chromosome evolution.

In this study, the first T2T gap-free reference genome of mulberry was assembled by combining high-coverage and accurate long-read sequence data from PacBio HiFi and Oxford Ultra-long sequencing. Strikingly, we report the full characterization of a polycentric genome, which is the only reported polycentric genome up to now. We further propose a suitable model for the chromosome fusion-fission cycle in *M. notabilis*. Our results shed light on the genome organization and chromosome evolution of this species, providing a scientific reference for further genetic studies and genetic improvement of mulberry.

## Results

### A T2T mulberry genome for *M. notabilis*, Mnot-SWU

**Figure 1:**
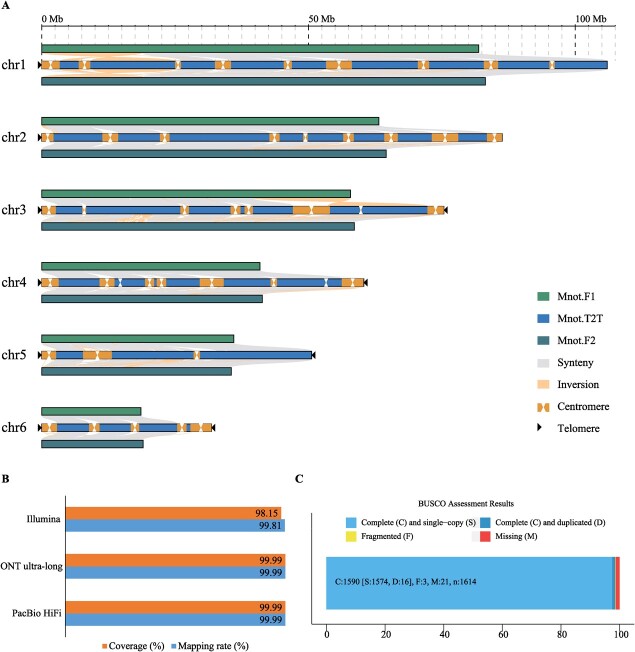
The gap-free reference genome of mulberry, Mnot-SWU. **A** Collinearity analysis among Mnot-SWU, Mnot.F1, and Mnot.F2. **B** Mapping rate and coverage of reads from different sequencing platforms. **C** BUSCO assessment for the completeness of genome assembly and gene model prediction with the embryophyte_odb10 dataset.

The estimated genome size was 389.35 Mb (Table S1, see online supplementary material). To develop a high-quality genome assembly for *M. notabilis*, we used various data generated via various sequencing platforms. In total, 15.06 Gb (~40× coverage) HiFi reads were generated by the PacBio Sequel II platform (Table S2, see online supplementary material), and 43.10 Gb (~110× coverage) ONT ultra-long reads were produced using the ONT platform (Table S3, see online supplementary material). The N50 length of the HiFi and ONT ultra-long reads was greater than 15.75 kb and 100.71 kb, respectively (Tables S2 and S3, see online supplementary material). Both reads obtained from HiFi and ONT ultra-long were used to perform primary assembly, and four sets of primary contig genomes were generated (Table S4, see onlinesupplementary material). The Hifiasm [25] produced the best assembly genome, which not only had N50, N90, and the largest contig 2–4 times longer than other versions of assembly genomes, but also contained the fewest contig numbers. Therefore, the assembly genome generated by Hifiasm was set as the backbone genome. Meanwhile, we generated 40.69 Gb (~100× coverage) of HiC sequencing data for anchoring the contigs to chromosomes (Table S5, see online supplementary material). After that, all contigs could be anchored to six chromosomes, and we generated a new version of the assembly genome with 32 contigs, including 26 gaps (Table S6, see online supplementary material). The other versions of genome assembly from the ONT reads were used to fill the gaps of the backbone genome. After filling all 26 gaps, we generated a gap-free reference genome, named Mnot-SWU, which contained six chromosomes with a total length of 410.45 Mb and the length of N50 reached 75.38 Mb (Table 1, Fig. 1A; Figs S1 and S2, see online supplementary material). Compared with the previously reported genome, the quality of the genome of *M. notabilis* was greatly improved (Table 1; Table S7, see online supplementary material). The new assembly also corrected some assembly errors in the previously reported genome, including misoriented, misassembled, and ambiguous regions (Fig. 1A). Finally, ten telomeres were identified using the conserved telomeric repeat (5’-CCCTAAA/TTTAGGG-3′) as a sequence query (Fig. 1A; Table S8, see online supplementary material).

**Table 1:** Characteristics of the *Morus notabilis* genome, Mnot-SWU.

**Genomic feature**	**Mnot-SWU**
The total size of assembled contigs (Mb)	410.45
Number of contigs	6
Contig N50 (Mb)	75.38
Gaps	0
Number of telomeres	10
BUSCOs (%)	98.51
Number of genes	27 413
LTR assembly index score	19.26

The quality of the Mnot-SWU genome was assessed in a variety of ways. First, all reads from three different sequencing platforms, such as PacBio HiFi, ONT ultra-long, and Illumina, were mapped to the Mnot-SWU genome, and all mapping rates reached 99%, indicating that the assembled genome was highly contiguous, with high accuracy (Fig. 1B; Table S9, see online supplementary material). A total of 27 413 gene models were predicted by multiple methods, and 98.51% of the 1614 reference gene sets from the embryophyte_odb10 database could be identified in the 27 413 gene model set (Fig. 1C; Table S10, see online supplementary material). When we annotated these coding genes, we found that a total of 94.90% genes could be aligned to public databases, including NR, Swiss-Prot, KEGG, InterPro, Pfam, and GO (Table S11, see online supplementary material). The LTR assembly index (LAI) of Mnot-SWU is 19.26 (Table 1). We also assessed k-mer-based quality estimates for Mnot-SWU genome, and the quality value of the genome is 40.24. When it comes to all chromosomes, the quality values are ranged from 39.18 to 41.72 (Table S12, see online supplementary material). Combining these results, the T2T Mnot-SWU genome presented here has the highest reliability and quality.

The T2T Mnot-SWU genome provides an unprecedented opportunity to identify all TEs, other repeat sequences and non-coding genes. As a result, a total of 242.97 Mb interspersed repeats were identified (Table S13, see online supplementary material), accounting for 59.20% of the assembly genome.

### Each chromosome of Mnot-SWU was split into several tandem repeat-rich intervals

**Figure 2:**
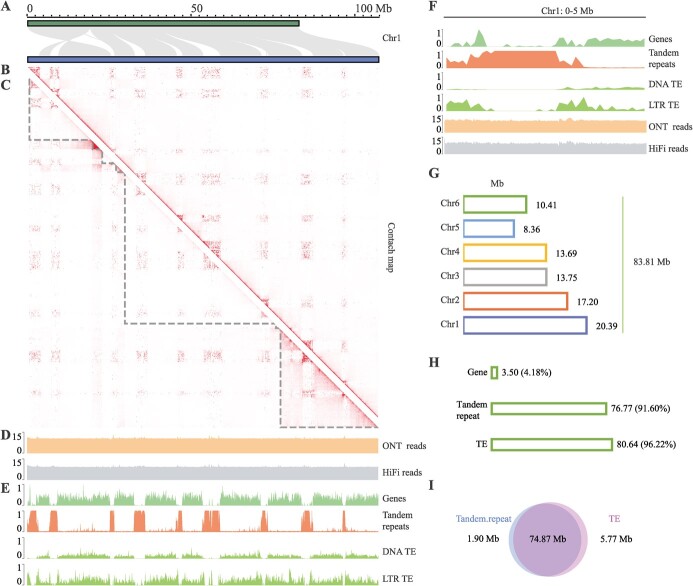
Chromosomes of Mnot-SWU were split into several repeat-rich regions by tandem repeats. **A** Detailed view of the collinearity of chr1 between Mnot-SWU (blue) and Mnot.vF1 (green). Gray lines indicate collinear regions of chr1 between the two genomes. Blank regions represent newly assembled regions in the Mnot-SWU genome that were not present in the previous genome. **B** Heatmap of chromosome interactions of chr1 based on HiC data in Mnot-SWU. **C** Heatmap of chromosome interactions of chr1 based on HiC data in the primary assembly by Hifiasm. Each triangular area with a gray dotted line represents a contig in the chr1 in the primary assembly. **D** Coverage of reads from ONT ultra-long (up) and PacBio HiFi (down) in chr1. A transformation (log2) was applied, and the y-axis gives the transformed values. **E** Chr1 detailed view showing the clustered genomic feature distribution. Windows sizes for genomic features distribution were set as 100 kb. From top to bottom, gene, tandem repeat, DNA type TE, and LTR type TE. **F** Zoomed-in view of Chr1 showing a 5-Mb region, near to the edge of the first contig of the primary assembly chr1. All values were calculated over 100-kb windows. **G** The size of the newly assembled regions on each chromosome in the Mnot-SWU genome. **H** Genomic features content of the newly assembled regions in the Mnot-SWU genome. **I** Comparison of tandem repeats and TEs in the newly assembled area.

Based on the collinearity results between Mnot-SWU and the other two previously reported genomes, Mnot.F1 [5] and Mnot.F2 [7], there were numerous unaligned regions on each chromosome, which were newly identified in Mnot-SWU (Fig. 1A). To clearly exhibit them, we redrew the collinearity of the longest chromosome (chr1, 106. 00 Mb) between the Mnot-SWU and the Mnot.F1 genome (Fig. 2A). Nine blank regions can be seen clearly in the results. The same phenomenon was found for five other chromosomes by collinearity analysis between genomes, with the number of blank regions ranging from three to nine (Fig. S3, see online supplementary material).

A surprising phenomenon emerged when we performed chromatin interaction analysis. In general, a typical chromatin interaction within euchromatin and heterochromatin in monocentric species exhibits large-scale compartmentalization and some degree of a telomere-to-centromere axis [26]. We also observed large-scale compartments in all chromosomes, but their numbers were not identical to those of a typical monocentric species (Fig. 2B; Fig. S4, see online supplementary material). For example, we also found nine large-scale compartments and another clustered axis in chr1 (Fig. 2B). The distribution pattern of the chromatin interaction in chr1 immediately caught our attention because this pattern was consistent with the previous collinearity analysis results of chr1 between Mnot-SWU and Mnot.F1 (Fig. 2A and B). The other five chromosomes showed the same characteristic (Figs S3 and S4, see online supplementary material).

The chromatin interaction of chr1 from the primary assembly genome was analysed, and the phenomenon was proved. As shown in Fig. 2C, the primary assembly chr1 contained seven contigs, and most of the new assembly regions (blank regions in Fig. 2A and clustered axis in Fig. 2B and C) in the Mnot-SWU genome were located inside one of the contigs. The HiFi reads and ONT ultra-long reads were evenly distributed within those newly assembled intervals (Fig. 2D), which also provides strong evidence that each of the newly assembled regions was contiguous and of high quality. These results were non-exceptional in the other chromosomes (Figs S5 and S6, see online supplementary material).

Notably, the distribution of genomic features also showed similarity patterns in each chromosome. First, *M. notabilis* genes were concentrated into nine intervals (Fig. 2E;Fig. S7, see online supplementary material). Most of these tandem repeats were identified in those newly assembled regions of the Mnot-SWU genome, and the coverage of tandem repeats reached 100% in most of the 100-kb bins of those intervals (Fig. 2E;Fig. S8, see online supplementary material). We also calculated the coverage of DNA and LTR transposable elements across 100 kb windows. Both showed patterns similar to those of genes (Fig. 2E; Fig. S9, see online supplementary material). All genes as well as DNA and LTR transposable elements were segmented into nine intervals by tandem repeats (Fig. 2E; Fig. S8, see online supplementary material). The zoomed-in view of chr1 showed a 0–5-Mb region, which was located at the edge of the first contig in the primary assembly chr1. The distribution pattern of genomic features in the edge of one contig showed no significant difference to that inside the same contig or in other contigs (Fig. 2F).

The distribution patterns of these genomic features and the newly assembled region across all six chromosomes prompted us to further analyse these interval features. We calculated the size of all newly assembled regions on all chromosomes, and a total of 83.81-Mb intervals were newly assembled in the Mnot-SWU genome (Fig. 2G). From chr1 to chr6, the size of newly assembled intervals was 20.39, 17.20, 13.75, 13.69, 8.36, and 10.41 Mb, respectively. The size of the newly assembled interval on each chromosome was positively correlated with the corresponding chromosome size, except for that in chr5. Subsequently, we focused on the composition of these newly assembled regions in all six chromosomes (Fig. 2H). A total of 3.50 Mb (4.18% of 83.81 Mb) of these newly assembled sequences were annotated as gene-related sequences, including UTR, exon, and intron. Regarding the other sequences, 76.77 Mb (91.60%) of newly assembled sequences could be classified as tandem repeat, and a total of 80.64 Mb (96.22%) of these sequences were regarded as TEs. We calculated the overlap between tandem repeats and TEs, and after comparing the positions of tandem repeats and TEs, we found that 74.87-Mb sequences, which occupied 97.53% of 76.77-Mb tandem repeats among the newly assembled sequences, were simultaneously defined as tandem repeats and TEs (Fig. 2I).

Based on the above results, each chromosome of *M. notabilis* was divided into several tandem repeat-rich intervals.

### *M. notabilis* is a species with repeat-based polycentric chromosomes

**Figure 3:**
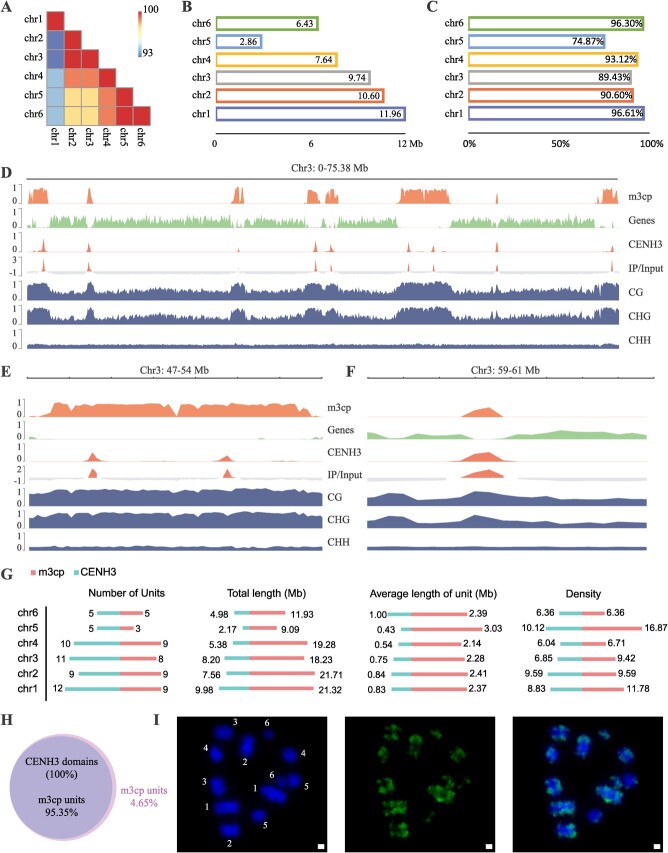
Features and genetic composition of repeat-based polycentromeres in *Morus notabilis*. **A** Heatmap of sequence identity of *m3cp* among all six chromosomes. **B** Total amount of *m3cp* repeats per chromosome. **C** The proportion of *m3cp* repeats located in newly assembled regions. **D** Global view of chr3 feature distribution, including the distribution of *m3cp* arrays, genes, CENH3 domains, ratio of IP and Input, and three types of 5mC methylation (CG, CHG, and CHH). All values were calculated across 100-kb windows. **E** Close-up view of chr3 showing a 7-Mb region with two centromere units, which are composed of two CENH3 domains and *m3cp* arrays. **F** Zoomed-in view of chr3 showing 2-Mb regions with CENH3 domain, whose length was longer than that of *m3cp* arrays. **G** Features of *m3cp* and CENH3 domains in *M. notabilis*. From left to right, number of units, total length, average length of unit, density of *m3cp* arrays and CENH3 domains per chromosome. **H** Comparison of intervals of *m3cp* arrays and CENH3 domains. **I** Chromosome location of *m3cp* by FISH. From left to right, DAPI, FITC, and merge.

The T2T genome of *M. notabilis* provides an excellent opportunity for us to study its centromeres and allows us to resolve complete centromeric repeat arrays in the Mnot-SWU genome. First, we analysed the sequence organization of centromeres in *M. notabilis*. The continuous and most abundant repeat unit was considered as the centromeric tandem repeat in each of the six chromosomes. As a result, we identified six centromeric tandem repeats in each chromosome, each with a length of 82 bp (Table S14, see online supplementary material). As the range of the global identity of those six centromeric tandem repeats was 93% to 100% (Fig. 3A), those repeats were named as *m3cp* and used for further analysis. We calculated the total amounts of the centromeric *m3cp* repeats per chromosome (Fig. 3B), which was positively correlated with corresponding chromosome size; the values were 11.96, 10.60, 9.74, 7.64, and 6.43 Mb in chr1, chr2, chr3, chr4, and chr6, respectively. Only 2.86 Mb *m3cp* arrays were annotated in chr5. The proportion of those repeats in the newly assembled regions was also assessed (Fig. 3C). A total of 96.91%, 90.60%, 89.43%, 93.12%, and 96.30% of *m3cp* repeats were newly identified in the Mnot-SWU genome in chr1, chr2, chr3, chr4, and chr6, respectively. The proportion of newly identified *m3cp* repeats was only 74.87% in chr5 of Mnot-SWU genome.

*Morus notabilis* is a polycentric chromosome species. We re-analysed the chromatin structure of *M. notabilis* and found that the genes of chr3 were concentrated into several regions along the entire chromosome (Fig. 3D). On the contrary, all centromeric *m3cp* repeats were located in the low-density gene intervals. Obviously, there were eight intervals that were enriched in the centromeric *m3cp* repeats, along with the chr3 chromosome. The range number of centromeric *m3cp* repeat-rich intervals in the other five chromosomes was from three to nine (Fig. S10 and Table S15, see online supplementary material). At the same time, we also carried out ChIP-seq experiments to determine the binding domain of centromeric histone H3 (CENH3) in chromosomes of *M. notabilis* (Fig. 3D; Fig. S10, see online supplementary material)*.* As shown in Fig. 3D, we detected nine clustered CENH3-binding domains, distributed across the chr3 chromosome. There was a long centromeric *m3cp* repeat array in the chr3: 47–54-Mb region, in which two CENH3 domains were detected (Fig. 3E). The length of most of the *m3cp* arrays was greater than that of CENH3 domain in all six chromosomes (Fig. 3D). Fig. 3F shows the zoomed-in view of other *m3cp* arrays, whose lengths were greater than that of the CENH3 domain. Note the 5mC methylation status of the entire chromosome (Fig. 3D, E and F). Remarkably, methylation in CG and CHG contexts was more pronounced for the *m3cp*-enriched regions than for other regions. Instead, we obtained a uniform pattern for mCHH at centromeric repeats.

The features of the centromeric *m3cp* repeats and CENH3 domains were compared in various ways (Fig. 3G). The total number of units of *m3cp* and CENH3 domains per chromosome ranged from five to nine, and five to twelve, respectively. Regarding the density of these units, they showed patterns similar to those of the unit numbers. Overall, the total length of *m3cp* arrays per chromosome (9.09–21.32 Mb) was greater than that of CEH3 domains (2.17–9.98 Mb). A similarity phenomenon was found for the average unit length, which ranged from 2.14–3.03 Mb for *m3cp* array and 0.43–1.00 Mb for CENH3 domains. The total number of *m3cp* arrays was 43, which is smaller than that of the CENH3 domain (52). Notably, 100% of CENH3 domains could be mapped to 93.55% of the *m3cp* arrays regions (Fig. 3H). We also conducted Fisher’s test for these two type intervals, obtaining a *P* value below 0.05; this indicates that genome-wide there was a significant association between *m3cp* repeats and CENH3 domains. In other words, the ChIP-seq results also proved that the *m3cp* repeats are centromeric tandem repeats and main CENH3-binding sites in *M. notabilis*. We further performed fluorescence *in situ* hybridization (FISH) to exhibit the distribution of *m3cp* repeats in all six chromosomes (Fig. 3I). As a result, we found multi *m3cp* repeat signals in all six chromosomes, suggesting that *m3cp* repeats are distributed in multiple positions in each chromosome. This leads us to infer that *M. notabilis* is a species with polycentric chromosomes.

### Features and genetic composition of the specific chr5 in *M. notabilis*

**Figure 4:**
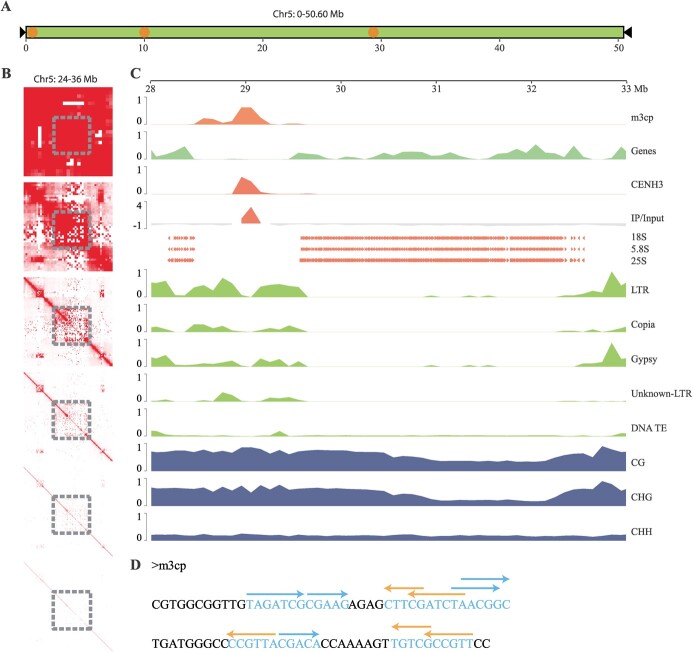
Features and genetic composition of the specific chromosome 5 of *Morus notabilis*. **A** Chromosome model of chr5. Black triangle denotes telomeres, orange circles indicate the approximate locations of the centromeres. **B** Contact maps for the assembled 24–36 Mb of chromosome 5. Gray squares indicate the 28–32-Mb region of chromosome 5. **C** Global view of genomic features distribution in the 28–32-Mb region of chr5. All values were calculated across 100-kb windows. **D** Patterns of dyad symmetries in *m3cp* repeat sequences. Blue and orange arrows represent positive or reverse directions, respectively.

The chr5 is a specific chromosome of *M. notabilis*. As mentioned above, only a total of 9.09 Mb *m3cp* repeat arrays were identified on chr5, whereas the values for the other five chromosomes ranged from 11.93 to 21.71 Mb (Fig. 3G). Considering that the size of chr5 is 1.6 times that of chr6, the total amount of *m3cp* repeats is only 76.19% of that in chr6. In addition, only three centromeres were identified on chr5 (Fig. 4A; Table S15, see online supplementary material), whereas the number of centromeres on the other five chromosomes ranged from five to nine. Both the average unit length and the density of *m3cp* repeat arrays and CENH3 domains were significantly different from those of the other five chromosomes (Fig. 3G).

**Figure 5:**
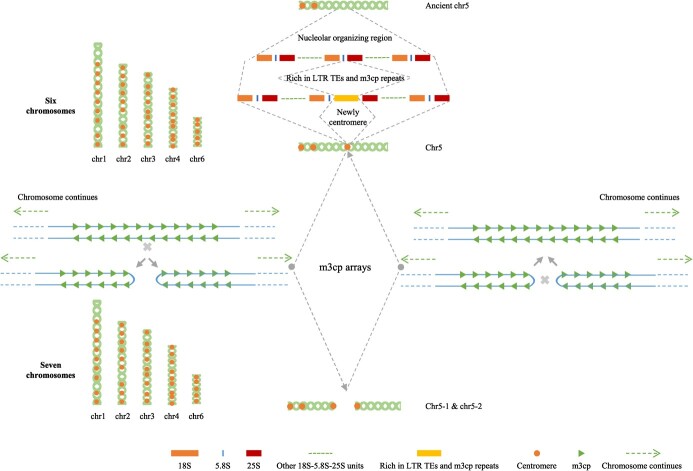
Proposed model for the chromosome fusion-fission cycle in *Morus notabilis*.

An abnormal chromatin interaction region was identified in chr5 chromosome. We performed chromatin interaction analysis on this chromosome based on the HiC data under different resolution levels, including 500, 250, 100, 50, 25, and 10 kb. Fig. 4B shows the contact map of 24–36 Mb of chr5. When the resolution was set as 250 kb, a region with little chromatin interaction emerged. As the resolution went from 250 to 10 kb, this region extended to both sides. We calculated the coverage of the genomic features in the entire chr5 across 100-kb windows; based on the results, LTR transposable elements were abundant in this region compared to other regions (Fig. S11, see online supplementary material). A centromere with a CENH3 domain was also found in this region. In addition, the rRNA genes that should have been distributed in one cluster were divided into two clusters by this centromere (Fig. 4C).

We re-analysed the sequence characteristics of the centromeric *m3cp* repeats; Fig. 4D shows the patterns of DNA dyad symmetry in the centromeric *m3cp* repeats. Notably, only lengths of dyad symmetry greater than 5 bp are shown for simplicity. When the dyad symmetry length was set as greater than 3 bp, 95.12% of the *m3cp* repeat length exhibited dyad symmetry structure (Table S16, see online supplementary material).

## Discussion

Here, we report a telomere-to-telomere gap-free and high-quality reference genome for mulberry species. The newly assembled genome is powerful not only for chromosome evolution studies but also for the genetic improvement of mulberry.

### The T2T genome of *M. notabilis* provides an unprecedented opportunity to study the mulberry genome

The first draft genome of mulberry was reported in 2013 [1]. Although some high-quality mulberry reference genomes have also been reported in recent years, they remained unassembled or unplaced [5, 7]. Genome assembly can be compared to a jigsaw puzzle with lots of pieces. To assemble a perfect genome, we need to pay more attention to the accuracy and continuity of sequencing. The successful assembly of near-complete or gap-free genomes by multiple sequencing strategy has been reported for some plant species in a new ear [27, 28]. In this study, the first mulberry T2T reference genome (Mnot-SWU) was achieved using a combination of new and cutting-edge sequencing technologies, including PacBio HiFi and Oxford Ultra-long sequencing. As a result, compared with previously reported mulberry genomes, a total of 83.81 Mb sequences were newly assembled in all six chromosomes. In addition, the telomeres are always composed of tandem repeats of relatively conserved microsatellite sequences in plants [29]. However, the conservative telomeric repeat has not been detected in the 3′ end of chr1 and chr2 in the Mnot-SWU genome. A similar phenomenon had also been found in some chromosomes of the T2T assembly of rice [11, 12], grapevine [30], bitter melon [19], and azalea [31]. Consider that other types of telomeric repeats had been identified in other plants, such as *Hyacinthella dalmatica* [32], and Aole species [33]. Therefore, we assumed that the reason may be that other types of telomeric repeats are also present in mulberry. The Mnot-SWU genome provides us with an unprecedented resource for the study of the entire mulberry genome.

### To date, *M. notabilis* is the only reported polycentric chromosomes species

The T2T reference genome produced here facilitated the first global accurate assessment of centromeres in plants because centromeres are always extremely difficult to assemble. Recently, centromeres have also been assembled in some other species, which have a near-complete genome, including rice [10–12], *A. thaliana* [13–15], banana [16], watermelon [17], barley [18], bitter melon [19], *B. rapa* [20], *R. tomentosa* [21], and strawberry [22]. All these species share a common trait, namely having only one centromere on each chromosome. In other words, they are monocentric species. In addition, high-quality genomes of three holocentric species have also been reported in detail. All holocentromeres were evenly distributed along the entire chromosomes of three *Rhynchospora* species [34]. After this, a new assembly of the 81.6-Mb centromere of pea chromosome 6 exhibited another type of centromere organizations, namely metapolycentromeres [35]. Real centromeric *m3cp* repeats and entire centromeres were also identified in *M. notabilis* because of the availability of the T2T reference genome assembled in this study.

Remarkably, *M. notabilis* is a species with polycentric chromosomes. To our knowledge, species with polycentric chromosomes have not been reported before. As mentioned above, the genomic features show an uneven distribution pattern along the chromosome length in monocentric species. For example, genes are generally concentrated toward telomeric regions, and repeats are often clustered nearby the centromeric regions [11]. As reported for holocentromeric chromosomes, all holocentromeres are evenly distributed along the entire chromosomes of three *Rhynchospora* species, and the main genomic features also exhibit a uniform distribution pattern along the entire chromosomes [34]. Regarding metapolycentromeres, an extended primary constriction was found in a chromosome, and multiple separate centromeric domains were found in the extended primary constriction [35]. By contrast, in *M. notabilis*, the main genomic features could be divided into several intervals along all six chromosomes.

Considering the distribution patterns of different types of centromeres, we assumed that the polycentromeric chromosomes represent an intermediate state between monocentric and holocentric chromosomes. Previous studies suggest that metapolycentromeric chromosomes also represent an intermediate state between monocentric and holocentric chromosomes [35, 36]. Compared with the metapolycentromeric chromosomes in pea, which have multiple separate centromeric domains in one primary constriction [35], we also found multiple CENH3 domains that were located in one centromere region in *M. notabilis*. However, centromere regions could be identified at multiple locations on the six chromosomes of mulberry.

We also focused on the mechanisms underlying polycentromere formation. As reported for rice, a new centromere domain arises in a nearby genomic region of chromosome 8 after the deletion of a part of an older centromere [37]. The mechanisms proposed for rice may explain the new formation of partially adjacent CENH3 domains. Segmental duplications are the reasons for the propagation of CENH3 domains in metapolycentromeres [35]. In addition, helitron elements mediated the transposition of centromeric repeats, which accelerates the spread of repeat-based centromeric chromatin in holocentric species [34]. Considering that there are multiple long centromere regions, which are far from each other in the chromosomes, we assumed that LTR-like transposons, which can mediate the translocation of large fragments or chromosome rearrangement, play important roles in the formation of polycentromeres. The insertion time of both intact *Copia* and *Gypsy* elements inside the centromeric regions was significantly later than that outside the centromeric regions, indicating that those elements are indeed more active in the centromeric regions compared to other regions (Fig. S12, see online supplementary material). However, to uncover the precise mechanism involved in polycentromere formation, further studies are necessary.

### Proposed model for the chromosome fusion-fission cycle in *M. notabilis*

One of the most intriguing questions to be addressed is the chromosome fusion-fission cycle in *M. notabilis* (Fig. 5). In this study, chromosome 5 exhibited specific characteristics that differed from those of the other five chromosomes in *M. notabilis* and the contact map analysis results suggested the presence of an abnormal chromatin interaction region (chr5: 28–32 Mb), indicating a fusion/fission site. It is worth emphasizing that there were two intervals with a clustered distribution of rRNA (18S, 5.8S, and 25S) genes. The phenomenon of a chromosome fusion-fission cycle in *M. notabilis* was discovered by cytology experiments, and FISH results suggested that the approximate region was located nearby the gene location of 25S rRNA [5]. Considering that genes of rRNA (18S, 5.8S, and 25S) are always clustered in nucleolar organizing regions (NORs) of one or more chromosomes [38], and that only two 25 s rRNA signals are marked on chromosome 5 of *M. notabilis* [5], we proposed that all those rRNA (18S, 5.8S, and 25S) genes are clustered in the ancient chromosome 5 (Fig. 5). As mentioned above, we assume that LTR retrotransposons play important roles in the polycentromere formation. Because the proportion of LTR transposons in the 28.44–29.56-Mb interval of chr5 was significantly higher (*P* < 0.01) than that of the DNA transposons, with the insertion of one large fragment rich in LTR transposons and *m3cp* repeats between the position of the 5.8S and 25S rRNA genes of the ancient chromosome 5, all rRNA gene copies that originally clustered in a single region were divided into two clustered intervals. Subsequently, due to high abundance of *m3cp* centromeric repeats in this region, a new centromere with a CENH3 domain was formed at the site of *m3cp* arrays. In *Rhynchospora species*, centromeric *Tyba* repeats play important roles in end-to-end fusion of chromosomes [34], a mechanism that well explains the chromosome number change from seven to six in *M. notabilis*. At the same time, due to abundant *m3cp* centromeric repeats, which contained more than 95% dyad symmetry sequences in a single *m3cp* repeat, the newly formed centromere also becomes a hot spot for chromosome fission. In this way, it is ensured that two newly formed chromosomes have their own centromere, thereby maintaining the stability of the cell cycles. The model proposed here not only explains the formation mechanism of new centromeres, but also clarifies the long-unsolved scientific question of the chromosome fusion-fission cycle in mulberry.

## Materials and methods

### Plant materials and sequencing

*Morus notabilis* was cultivated under natural conditions in the mulberry breeding center in Beibei, Chongqing, China. Fresh young leaf tissue was collected and immediately frozen in liquid nitrogen, and high-quality DNA was isolated from fresh young leaves using the CTAB method and assessed with the NanoDrop One spectrophotometer (NanoDrop Technologies, Wilmington, DE, USA). The DNA quantity was measured with a Qubit 3.0 Fluorometer (Life Technologies, Carlsbad, CA, USA).

For SMRT sequencing, a standard HiFi library was prepared according to the SMRTbell Express Template Prep Kit 2.0 manual (Pacific Biosciences, CA, USA). The genome of *M. notabilis* was sequenced using the PacBio Sequel II system (Pacific Biosciences, Menlo Park, CA, USA). For ONT ultra-long sequencing, the standard library was prepared using the SQK-ULK001 kit, following the standard protocol. The purified library was sequenced using a PromethION sequencer (Oxford Nanopore Technologies, Oxford, UK). For HiC sequencing, genomic DNA was deep sequenced using Illumina HiSeq X Ten platforms in 150-bp PE mode according to the standard Illumina (Illumina, San Diego, CA, USA) protocols. For the proper annotation of the *M. notabilis* genome, total RNA from six different tissues (leaf, root, bud, bark, fruit, flower) was isolated using the RNAprep Pure Plant Kit (QIAGEN, Beijing, China). After assessment and measurement, the library was prepared according to the Oxford Nanopore (SQK-PCS109) cDNA-PCR Sequencing Kit and sequenced according to the standard protocol of the PromethION sequencer.

### Genome *de novo* assembly and quality assessment

The genome size of *M. notabilis* was estimated by k-mer frequency analysis with GenomeScope (v2.0) [39], after counting k-mers with Jellyfish (v2.3.0) [40]. Multiple strategies based on different algorithms were used for the *de novo* assembly of the genome and four sets of primary contig genomes were generated. The software package CCS (v6.4.0, https://github.com/PacificBiosciences/ccs) was used to filter consensus reads with default parameter. For HiFi sequencing, high-quality HiFi reads were subjected to assembly using Hifiasm (v0.16.1) with default parameters to generate a draft genome [25]. The reads obtained from ONT ultra-long sequencing were filtered and only high-quality reads were used for further assembly. NextDenovo (v2.5.0, https://github.com/Nextomics/NextDenovo) was applied for assembly, using the parameters ‘read_cutoff = 1k, blocksize = 1g, nextgraph_options = -a 1’. Necat (v0.0.1) [41] was used to assemble the genome with default parameters. Flye (v2.9) was employed to assemble the genome with ‘-i 3 -m 10000’.

After the initial assembly, Racon (v1.4.21) [42] and Pilon (v1.23) [43] were used to correct and polish the contigs for three rounds with ONT reads and Illumina PE reads to generate the final contigs. All Hi-C data were cleaned using fastp (v0.21.0) to generate high-quality clean data [44]. Subsequently, only valid interaction pairs of Hi-C data were obtained by HiCUP (v0.8.0, https://www.bioinformatics.babraham.ac.uk/projects/hicup/) for chromosome-level assembly. Then, ALLHiC (v0.9.8) [45] and Juicebox (v1.11.08) [46] were used to cluster, order, and orient the contigs. The assembly genome generated by Hifiasm was set as the backbone genome, and the ONT genome was used to fill the gaps present in the backbone genome. Telomeric reads from ONT were aligned to the genome using Winnowmap (v2.03) [47] and the obtained telomeric sequences (CCCTAAA/TTTAGGG) were patched for the telomere-missing chromosomes. Subsequently, the genome was corrected again by aligned HiFi reads to the genome, using Winnowmap (v2.03) [47]. Finally, a heatmap of genomic interactions from the T2T genome was produced with HiCExplorer (v3.6) [48].

The T2T genome completeness of *M. notabilis* was assessed using BUSCO (v5.2.2) with the embryophyte_odb10 database [49]. The contig N50 length was applied to reveal the continuity of the genome, and genome accuracy was evaluated. All reads generated by Illumina were mapped to the genome using BWA (v0.7.17) [50]. The PacBio HiFi and ONT reads were mapped to the genome with minimap2 [51], and LAI index [52] was calculated to assess the assembled genome.

### Gene and repeat annotations

Repetitive elements were identified by two strategies: *de novo* and structural signature-based ones. The *de novo* repeat library was constructed using RepeatModeler (v2.0) [53] and EDTA (v2.1.0) [54], and RepeatMasker (v4.0.9) was employed to annotate the interspersed repeats in the genome. Gene annotation was performed by combining various methods, including *ab initio*, homology-based and RNE-seq data-supported approaches. Two *ab initio* gene predictors, Augustus (v3.3.2) [55] and GlimmerHMM (v3.0.4) [56], were used, and BUSCO (v5.2.2) [49] was applied to obtain the training sets for *ab initio* gene predictors. For homology-based gene prediction, we selected *A. thaliana* [14], *M. notabilis* [5, 7], *M. yunnanensis* [7], *M. alba* [8], *M. indica* [9], and *Cannabis sativa* (https://plants.ensembl.org/Cannabis_sativa_female/Info/Annotation/#assembly) using Exonerate (v2.2.0, https://github.com/nathanweeks/exonerate).

We employed MAKER (v2.31.10) for the whole genome annotation pipeline, and IsoSeq reads from the ONT platform were sequenced by NanoFilt (v2.8.0, https://github.com/wdecoster/nanofilt), Pychopper (v2.7.2, https://github.com/epi2me-labs/pychopper), Racon (v1.4.21) [42], and minimap2 [51] to generate .bam files. The RNAseq reads were filtered by fastp and aligned to our assembly genome using hisat2 (v2.1.0) [57] to generate other .bam files. Subsequently, StringTie (v2.1.4) [58] and TransDecoder (v5.1.0, https://github.com/TransDecoder/TransDecoder) were used to predict gene models. Finally, all data were integrated to generate the final set of gene models using MAKER (v2.31.10) [59].

We further predicted the functions of genes according to the following methods: All coding genes were searched against other databases, including the KEGG [60], InterProScan [61], Swiss-Prot [62], Pfam [63], and GO [64] databases. The tRNAscan-SE (v2.0.9) [65] was employed to identify tRNA genes, and RNAmmer (v1.2) [66] and INFERNAL (v1.1.4) [67] were used to predict rRNAs, snRNAs, and miRNAs in the genome.

### Genome and gene collinearity

The function Nucmer, implemented in Mummer (v4.0.0beta2) [68], was used to perform comparisons between genomes with default parameters. Subsequently, the delta-filter command was carried out with parameters ‘-r -q’ to obtain one-to-one alignment block regions. SyRI (v1.6) [69] was used to identify syntenic regions between those genomes, and GenomeSyn (v1.2) [70] was applied to visualize genome collinearity. The python version of MCscan pipeline implemented in jcvi (v1.1.19) [71] was used to perform and visualize gene collinearity with default parameters.

### Detection of centromere and telomere sequences

The telomere sequence (5’-CCCTAAA/TTTAGGG-3′) was searched directly using seqkit (v2.3.1) [72]. Tandem repeats in the genome were identified by the Tandem Repeats Finder with the parameters ‘2 7 7 80 10 50 500’ [73]. After those repeats were clustered, we calculated the abundances of the corresponding repeats. The continuous and most abundant repeat unit was considered as the centromeric tandem repeat.

### Chromatin immunoprecipitation

The procedures for ChIP were carried out as described elsewhere [74]. Fresh young leaves were collected and immediately frozen in liquid nitrogen. The samples were fixed in 4% formaldehyde for 25 min, and the chromatin was sonicated and quality-controlled. The CENH3 antibody (N terminus) was prepared, and for ChIP, the sonicated chromatin was incubated overnight with 2 μg CENH3 antibody at 4°C. Subsequently, Dynabeads Protein G (Invitrogen, Carlsbad, CA, USA) was used to capture the target antibody, and bound chromatin was eluted, de-crosslinked, and precipitated. The ChIP-seq library was prepared with the TruSeq ChiIP library preparation kit (Illumina, San Diego, CA, USA) and sequenced on an Illumina NovaSeq6000 in 150-bp PE mode. No-antibody inputs were used as controls.

### ChIP-seq analysis and the completeness of centromeres on chromosomes

We used BLAST [75] to align the *m3cp* satellite repeats in mulberry to each reference chromosome, with parameters ‘evalue = 1e-5, and –task blastn-short’. Subsequently, we used BEDtools (v2.30.0) [76] to merge all adjacent *m3cp* monomers situated at a maximum distance of 50 000 bp with the parameter ‘-d 50 k’ to eliminate the gaps due to fragmented *m3cp* arrays. Then, the boundary of *m3cp* arrays was extended again with 500-kb-long windows until no *m3cp* repeats were found. The raw sequencing reads of CENH3 ChIP-seq were trimmed by Cutadapt (v4.2) [77], and BWA (v0.7.17) [50] was used to align the trimmed reads to the *M. notabilis* genome. All read duplicates were filtered to generate the final BAM file. The bamCompare and bamCoverage tool implemented in deeptools (v3.5.1) [78] was used to convert BAM files into BIGWIG coverage tracks with the parameters ‘--binSize 50, --normalizeUsing RPKM’. We used MACS (v3.0.0b1) [79] to identify the CENH3 signals by comparing the ChIPed and input data with parameters ‘-broad -g -broad-cutoff 0.1’. Subsequently, BEDtools (v2.30.0) [76] was applied to merge all adjacent peaks situated at a maximum distance of 50 000 bp with the parameter ‘-d 50 k’ to eliminate the gaps due to fragment *m3cp* arrays or TEs insertion. To filter negative results, only a merged region with more than five peaks was retained. After this, the boundary was extended to both flanking sides at 500-kb-long windows until no ChIP-seq peaks were found [11]. Both boundary of *m3cp* arrays and CENH3 domains were used to define the boundary of centromeres.

### Methylation analysis

First, nanopore reads were generated using base-calling by Guppy (v6.0.1) and re-squiggled using Tombo (v1.1.5, https://github.com/nanoporetech/tombo) according to the recommendations, creating an index and storing the raw signal alignments. Subsequently, cytosine methylation was analyzed by detecting the frequency of 5mC (CG, CHG, and CHH) from the nanopore reads using Tombo (v1.1.5, https://github.com/nanoporetech/tombo) with the alternative model. The frequencies of methylation for the cytosine position were calculated by the number of methylated reads/the number of methyl-free reads.

### Fluorescence *in situ* hybridization

Mitotic chromosomes were prepared according to the methods described in our previous report [5]. The best slides were chosen for FISH. The *M. notabilis* chromosomes were hybridized with digoxigenin-labeled *m3cp* oligo-probe synthesized by TsingKe (Beijing, China), and FISH was performed as described previously [5]. The final images were processed using the Adobe Photoshop CS6 software.

## Acknowledgments

We thank André Marques (Max Planck Institute for Plant Breeding Research) for his valuable comments on ChIP-seq analysis. We thank Jianming Zeng (University of Macau), and all the members of his bioinformatics team, biotrainee, for generously sharing their experience and codes. We thank Yangqin Xie for his suggestions. We thank Tian Li for helping to deposit the data. We also thank all members of our group.

This project was supported by the National Natural Science Foundation of China (32101544), and the Chongqing Research Program of Basic Research and Frontier Technology (cstc2021yszx-jcyj0004).

## Author contributions

Conceptualization, B.M. and N.H.; funding and resources, B.M., N.H; data production, formal analyses, investigation, and visualization, B.M.; experimentation, H.W., J.L., L.C., X.X.; sample preparation, B.M., H.W., J.L., X.X., W.W., Z.Y., J.Y., Y.L; writing, B.M.; review and editing: N.H. All authors read and approved the final manuscript.

## Data availability

All the raw sequencing data and genome assembly generated for this project are deposited at Nation Genomics Data Center under BioProject no. PRJCA015883, and the genome assembly and annotations are also deposited at MorusDB (https://morus.swu.edu.cn). All the materials in this study are available upon request.

## Conflict of interest statement

No conflicts of interest declared.

## Supplementary data

Supplementary data is available at *Horticulture Research* online.

## Supplementary Material

Web_Material_uhad111
