# Supplementary figures and images for: The gap-free genome of mulberry elucidates the architecture and evolution of polycentric chromosomes
Source: Hortic Res. 2023 May 31;10(7):uhad111. doi: 10.1093/hr/uhad111 (PMC10541557; doi:10.1093/hr/uhad111)

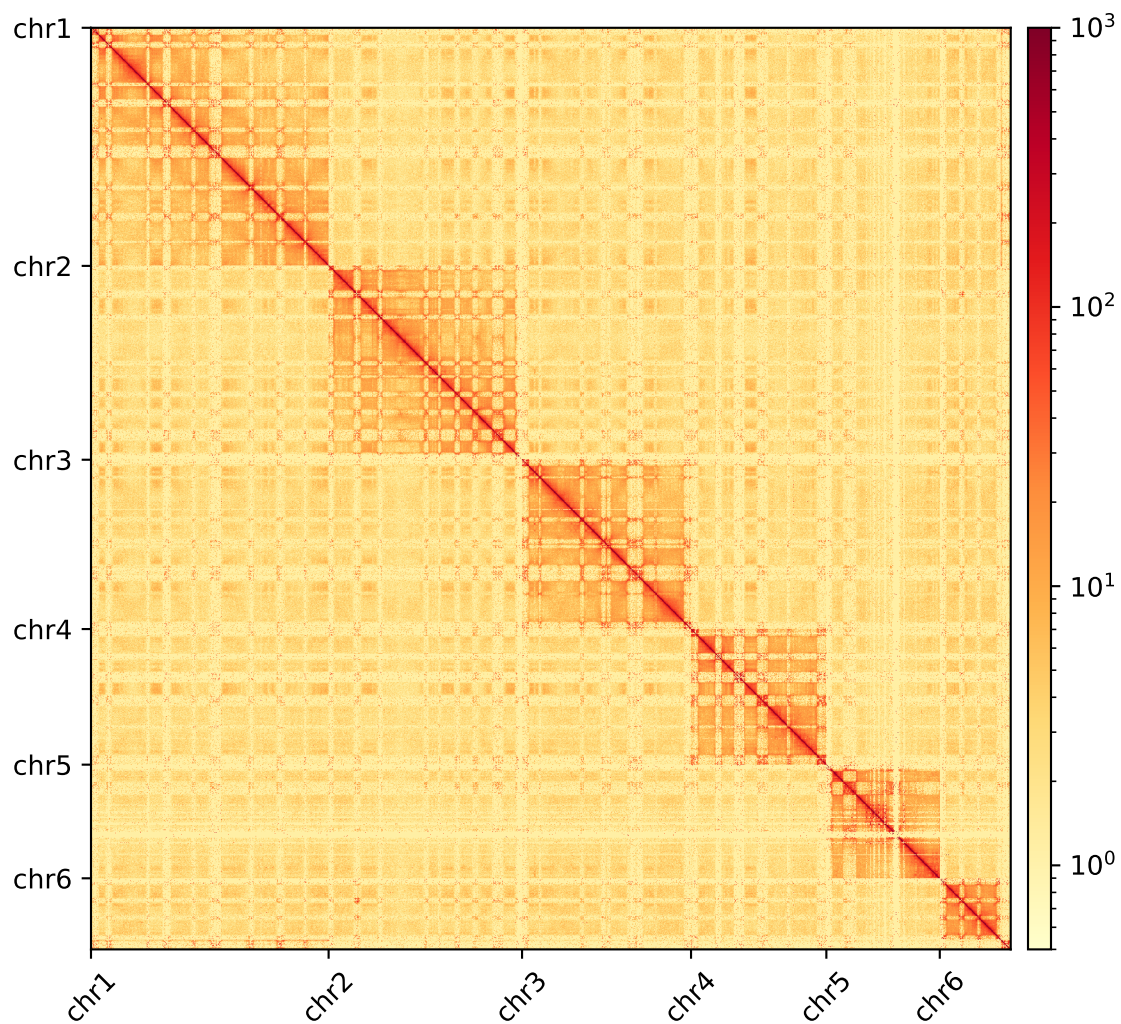

Supplement: Web_Material_uhad111 [file web_material_uhad111.zip › Figure_S1.pdf]

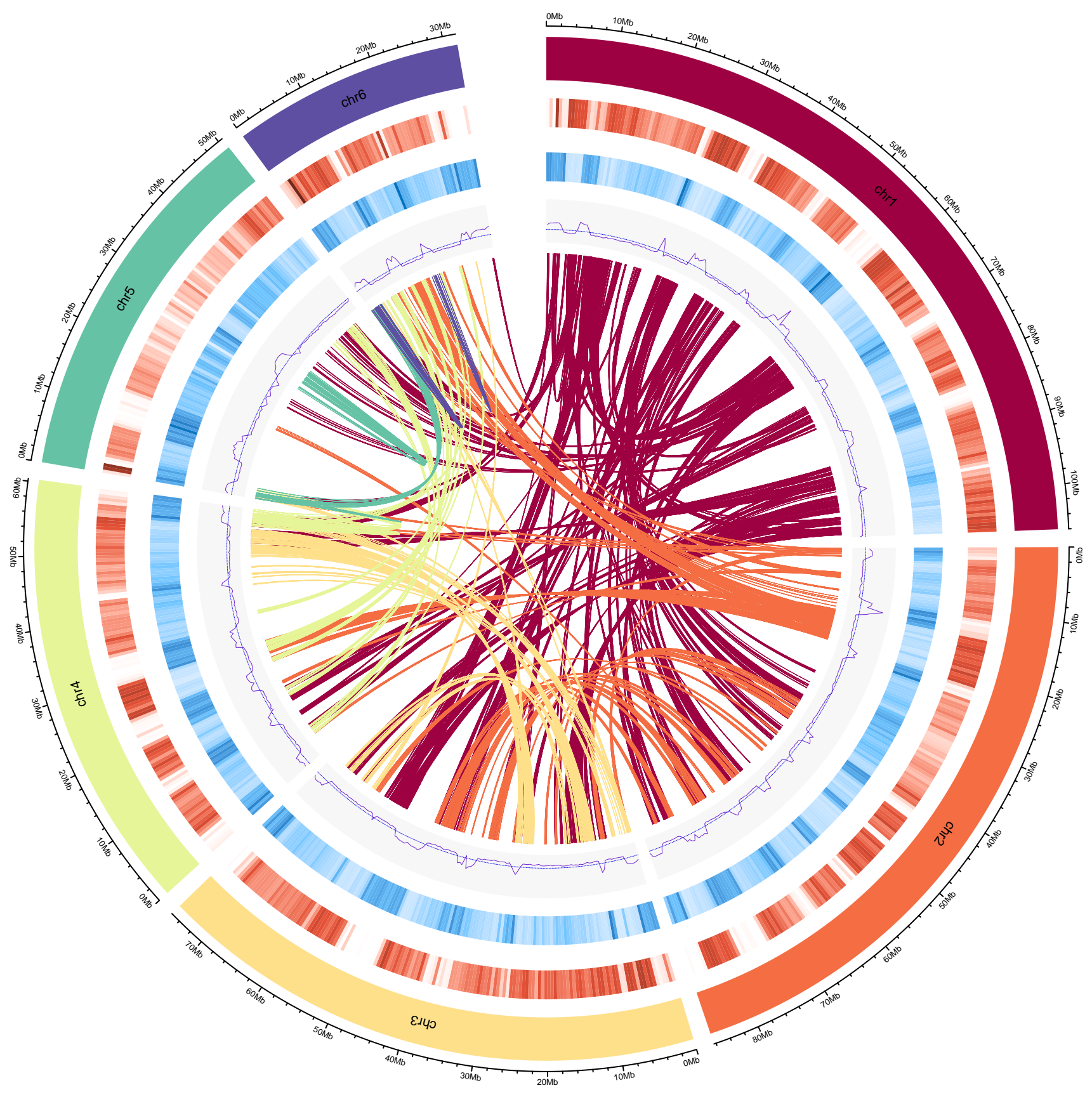

Supplement: Web_Material_uhad111 [file web_material_uhad111.zip › Figure_S2.pdf]

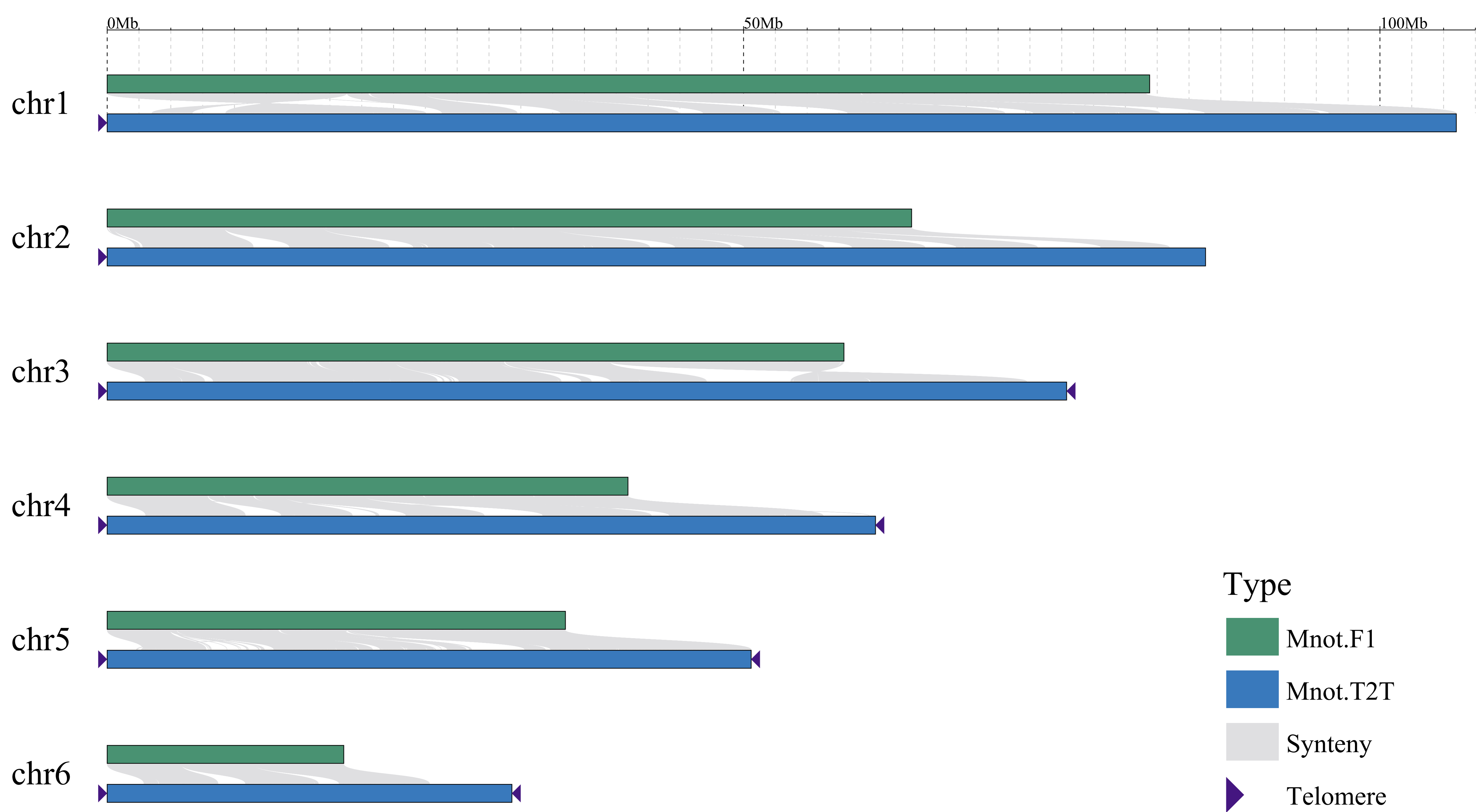

Supplement: Web_Material_uhad111 [file web_material_uhad111.zip › Figure_S3.pdf]

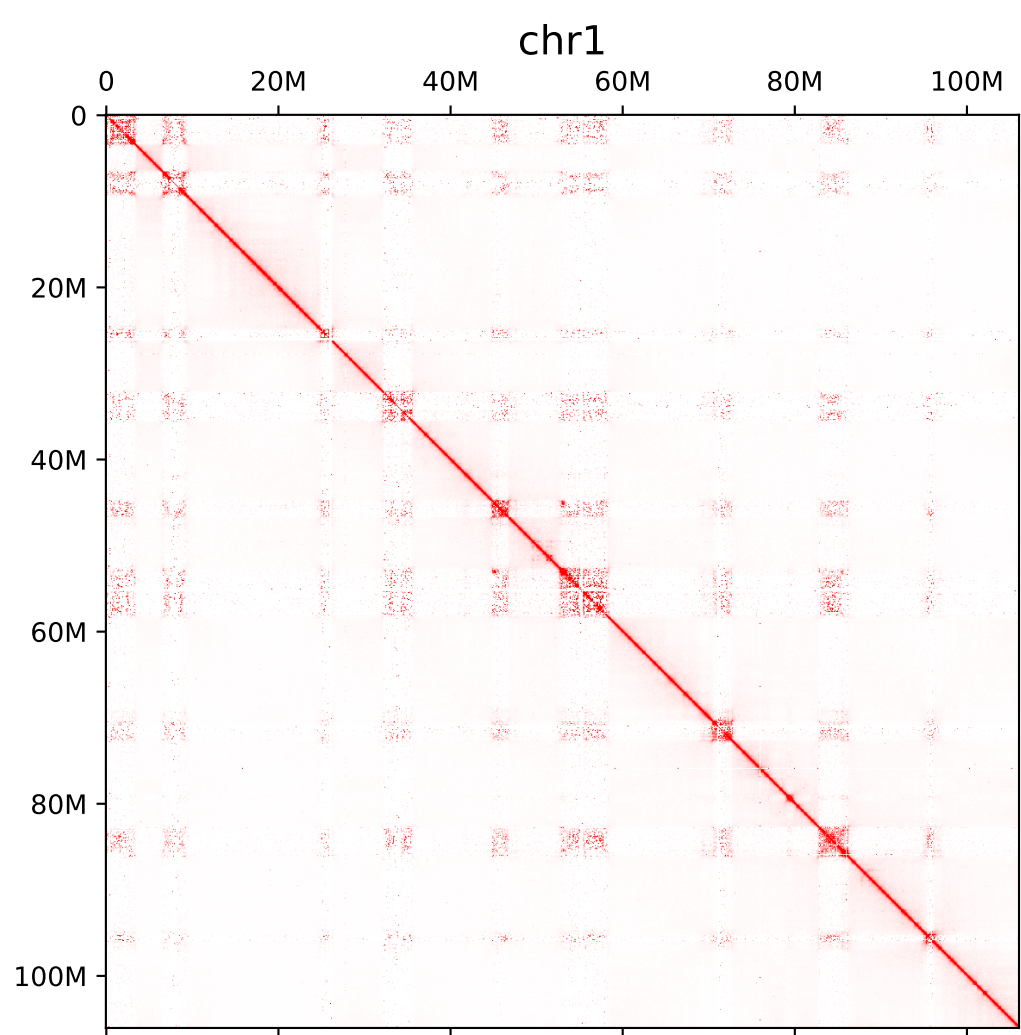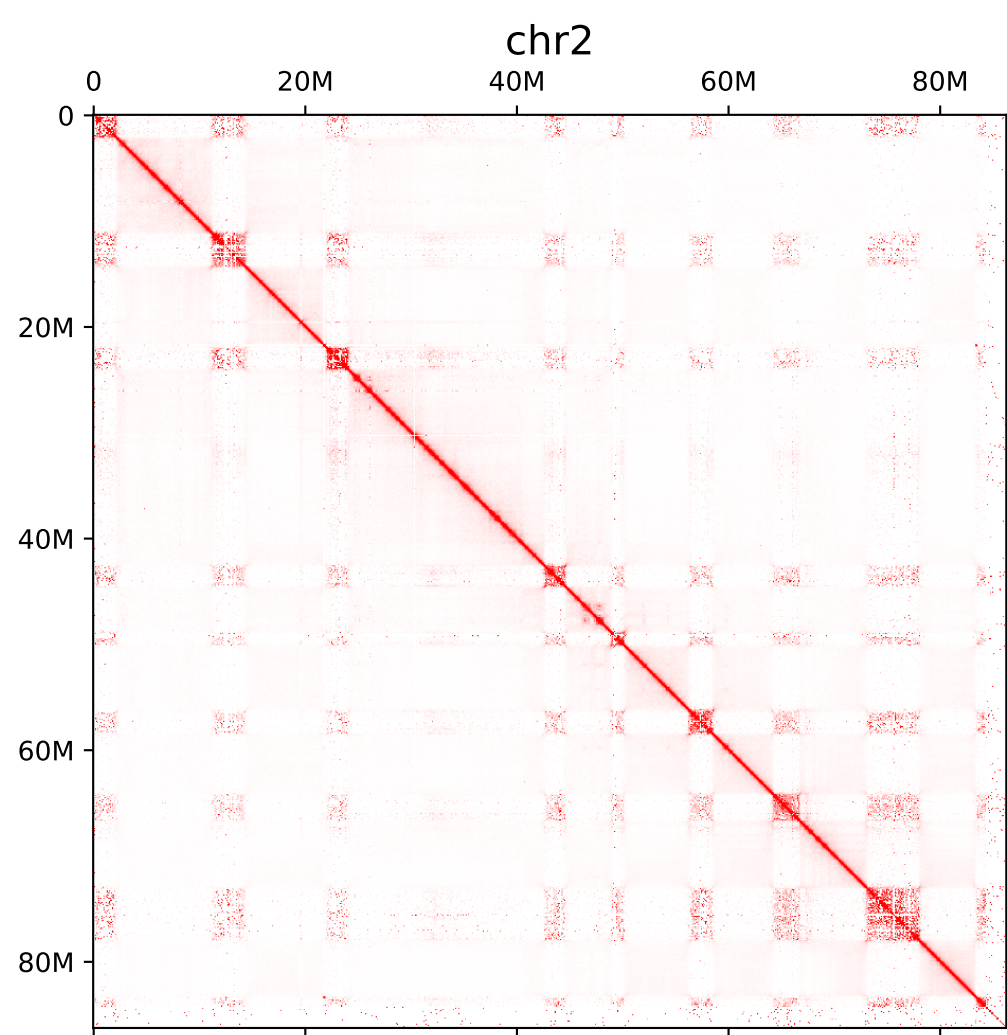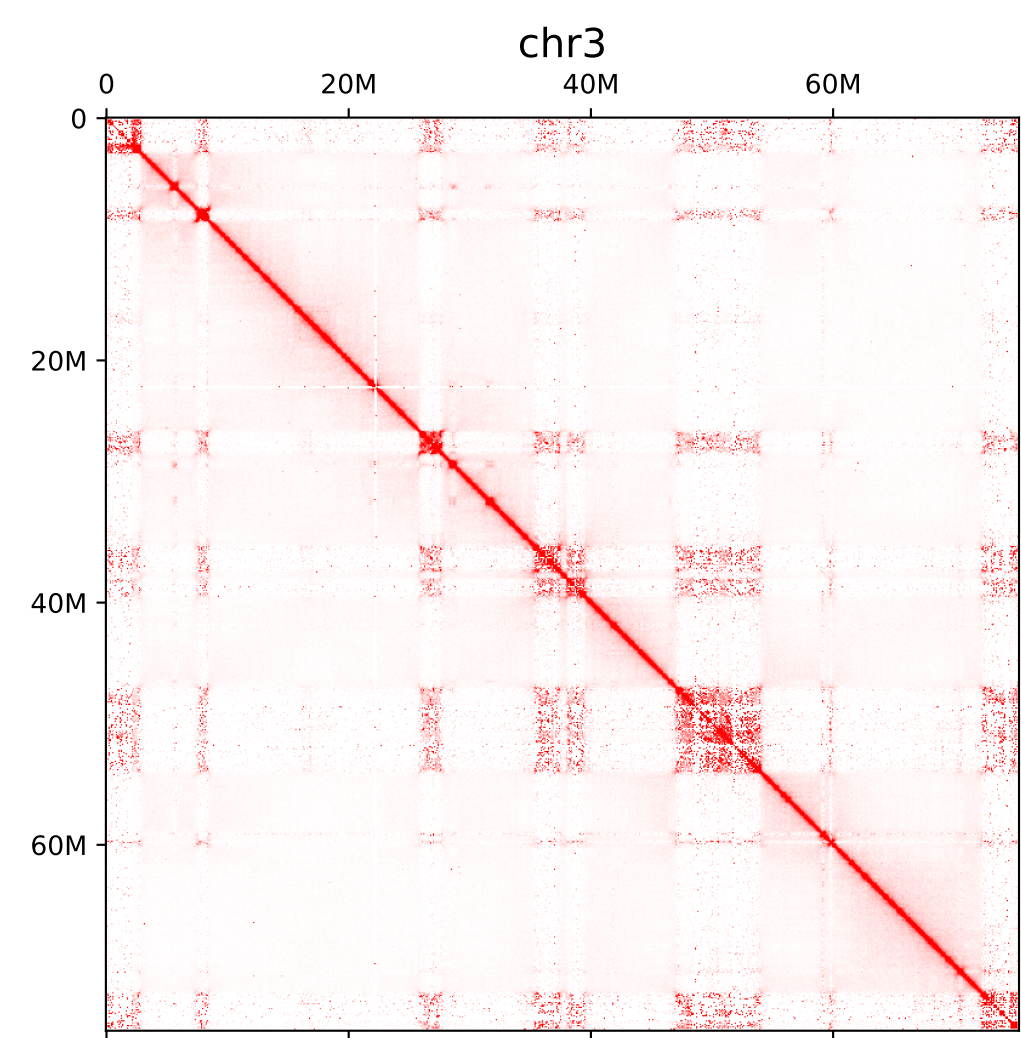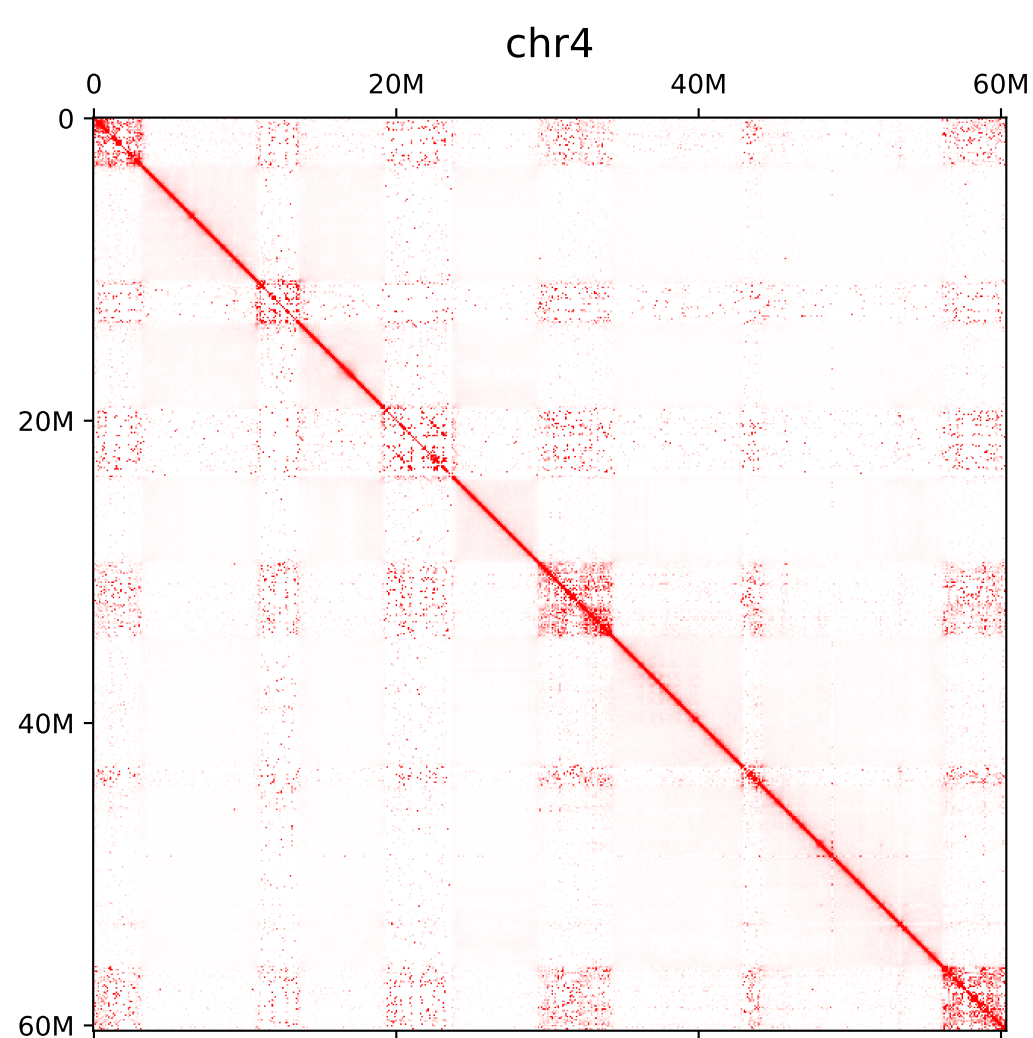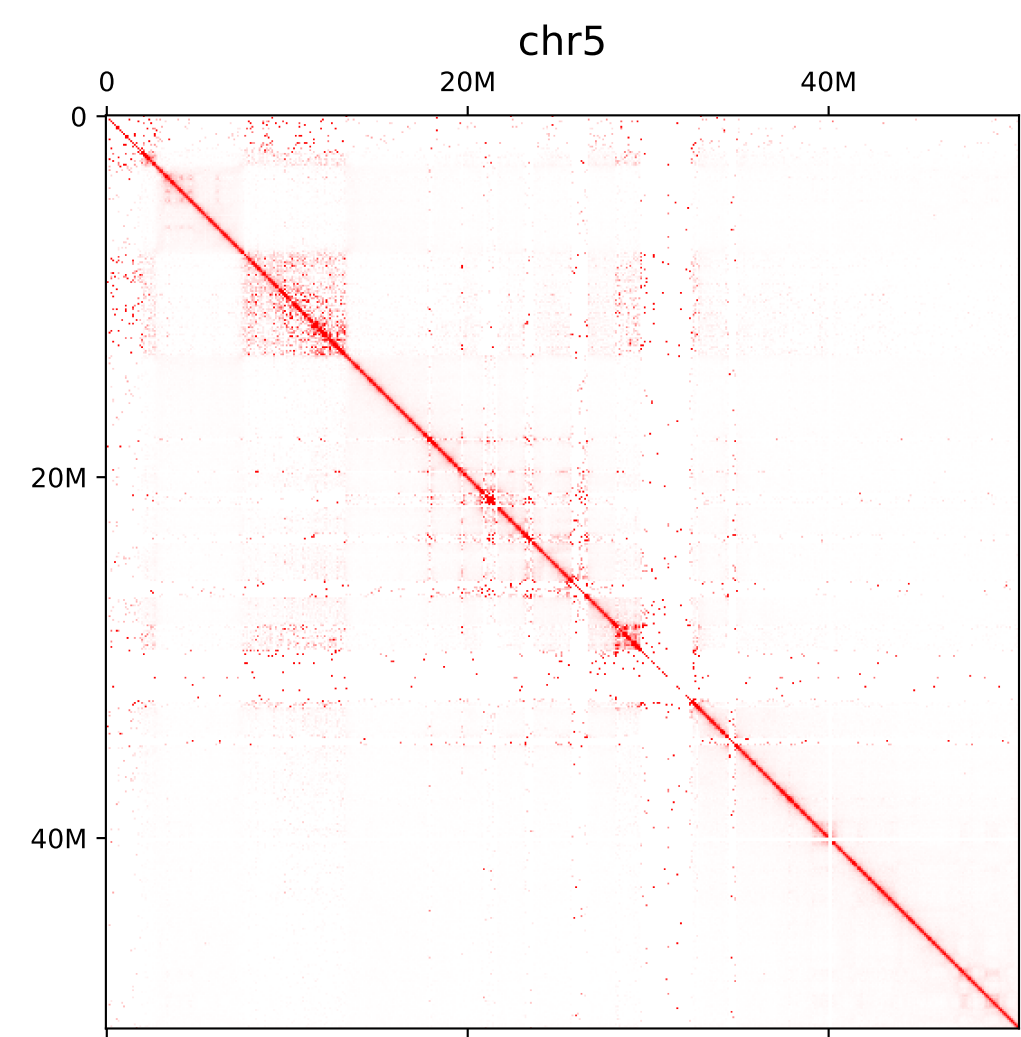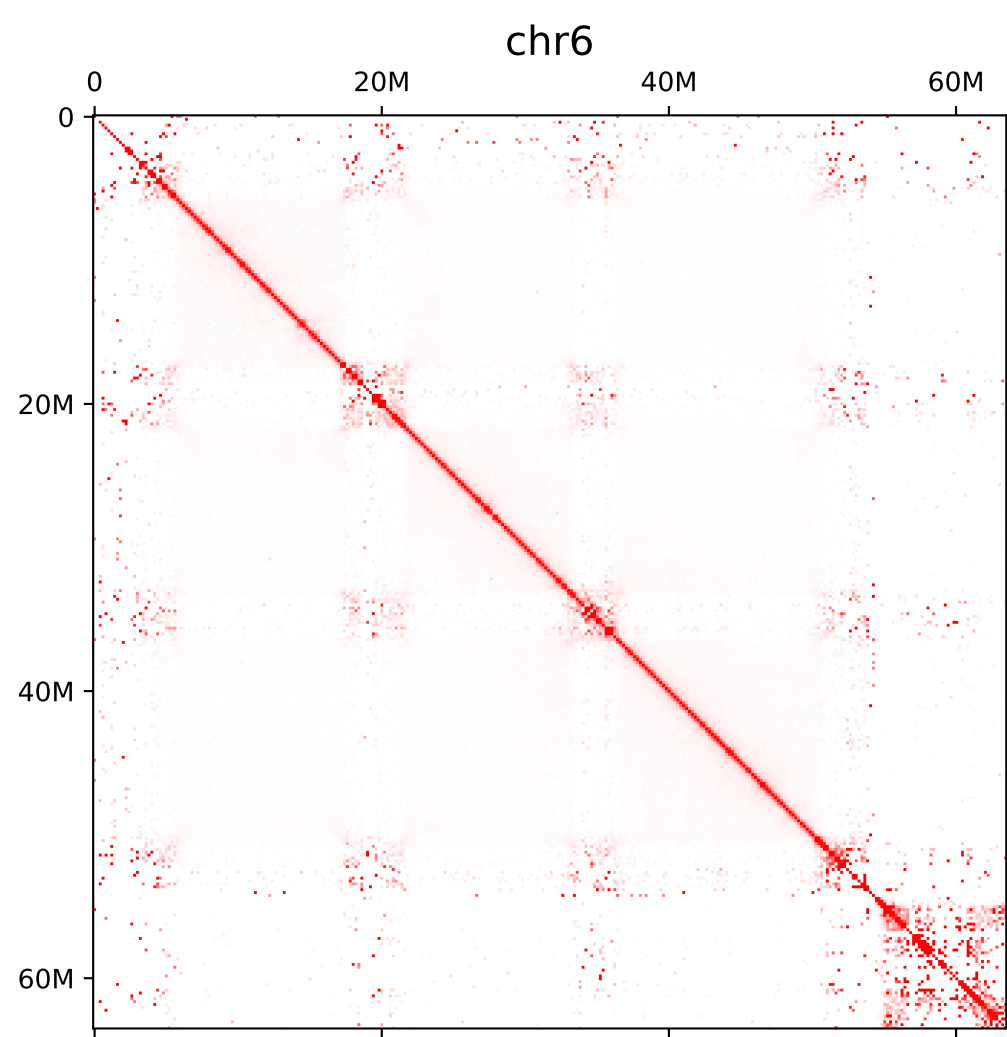

Supplement: Web_Material_uhad111 [file web_material_uhad111.zip › Figure_S4.pdf]

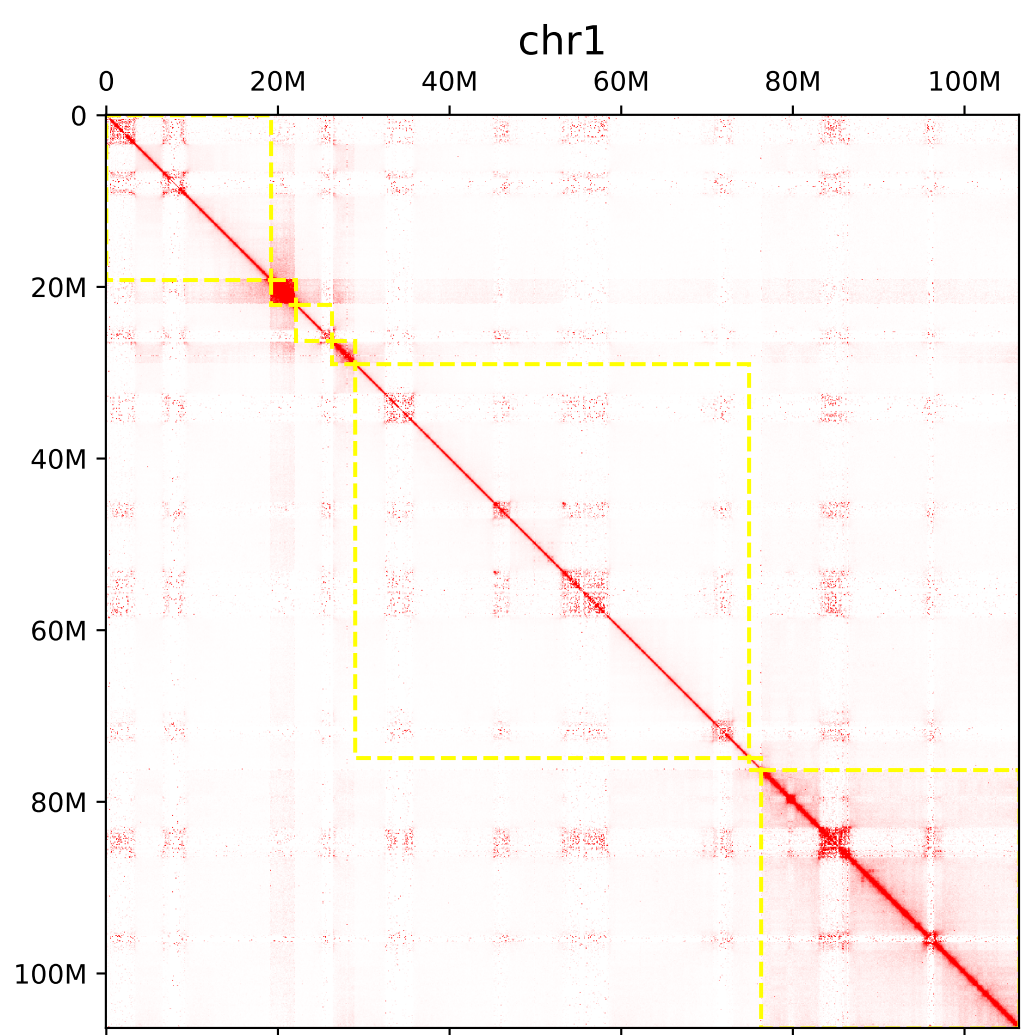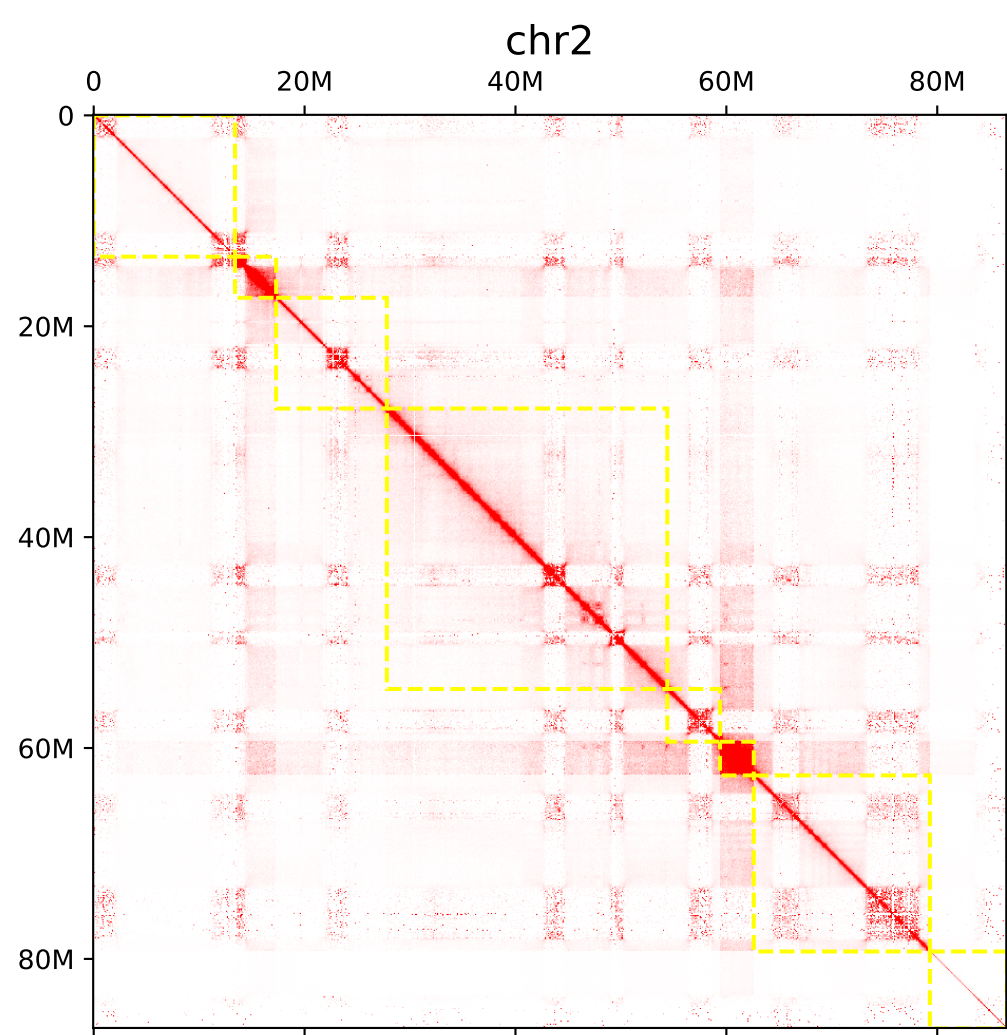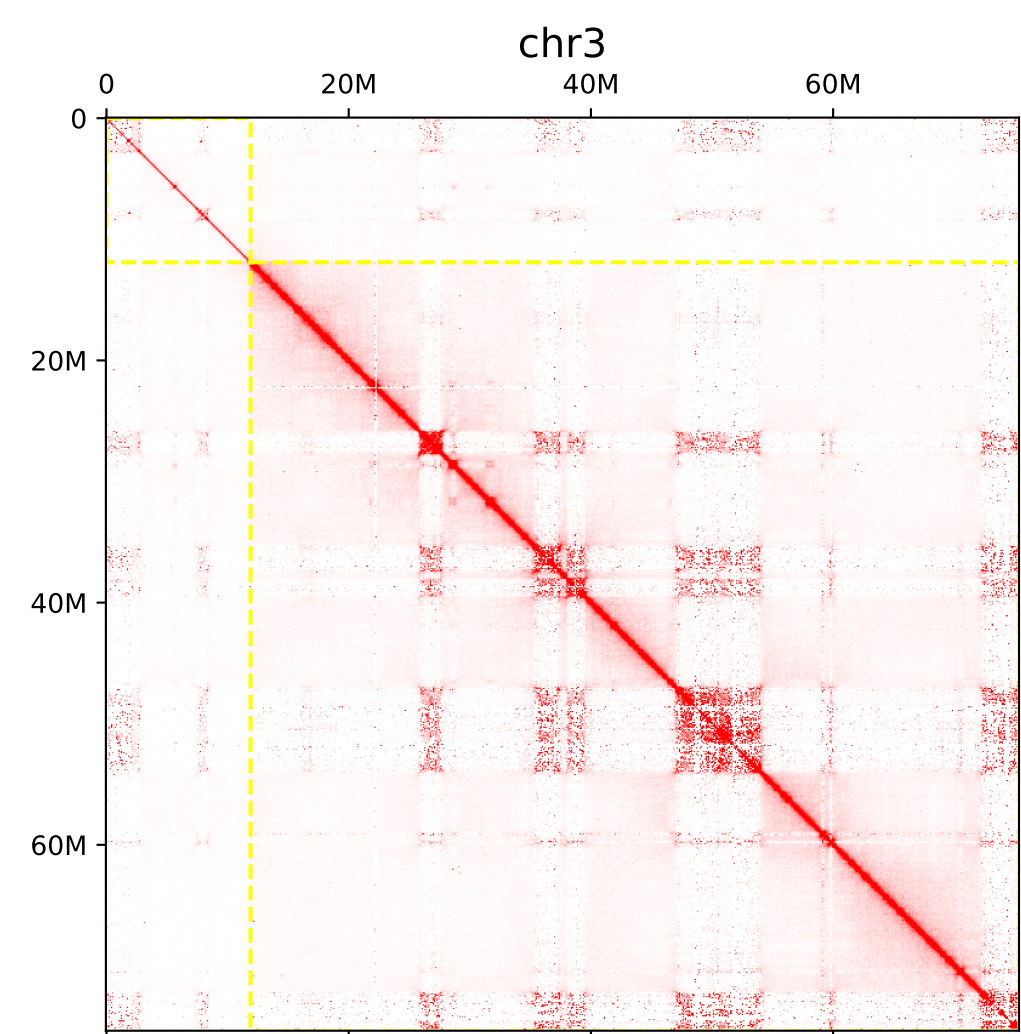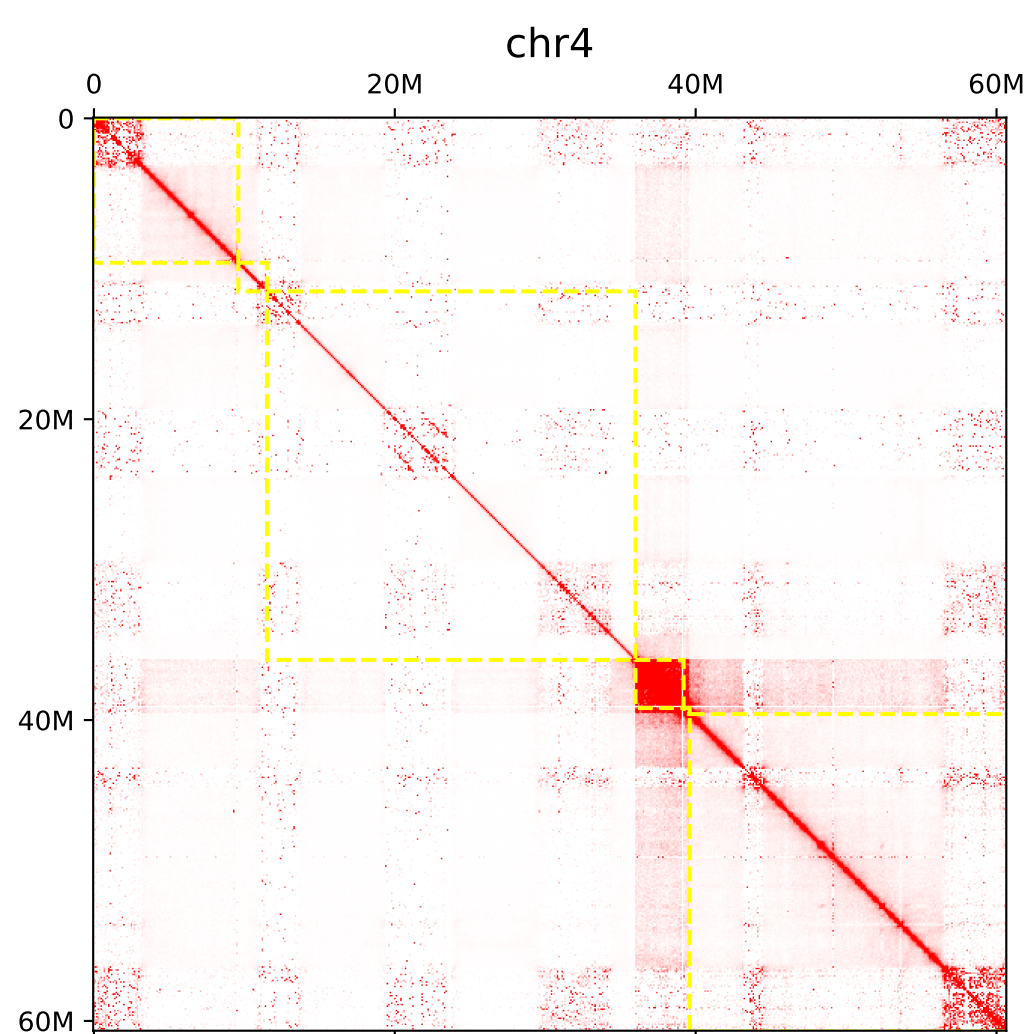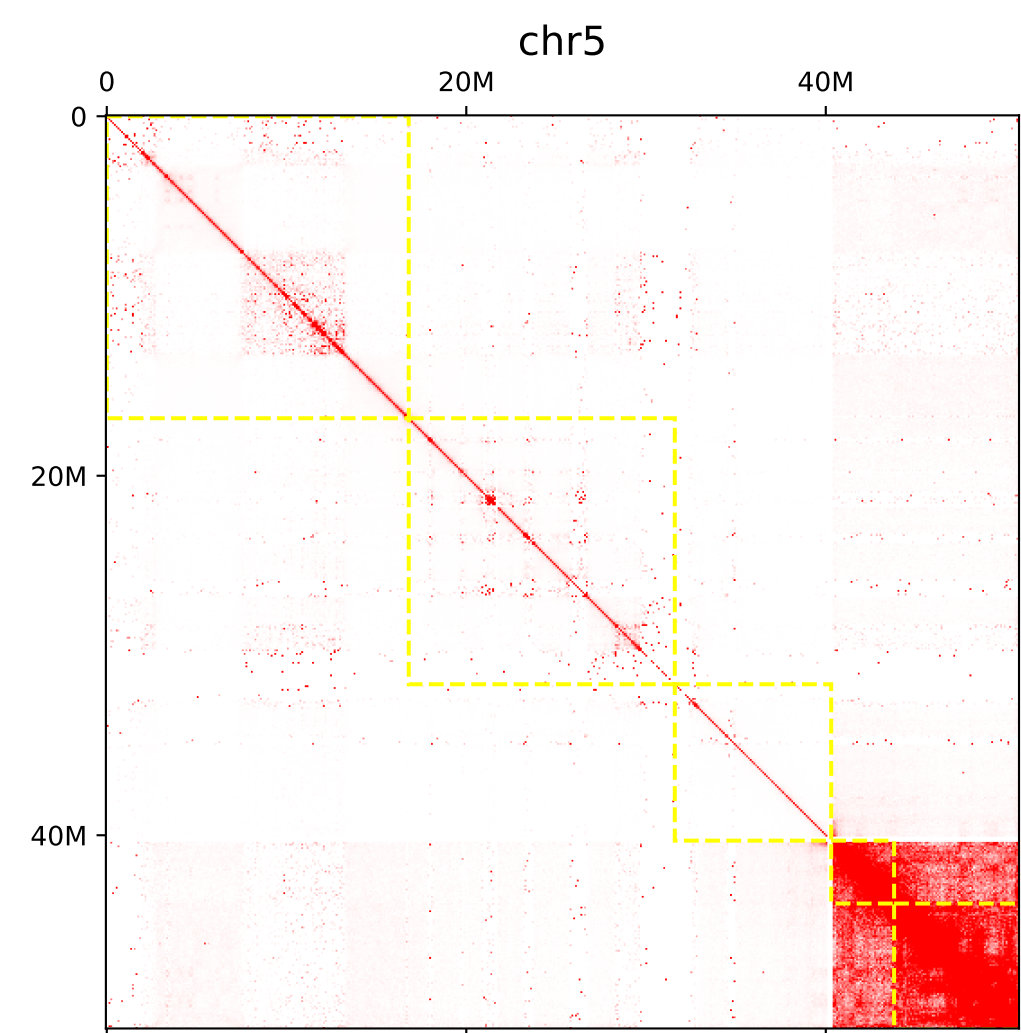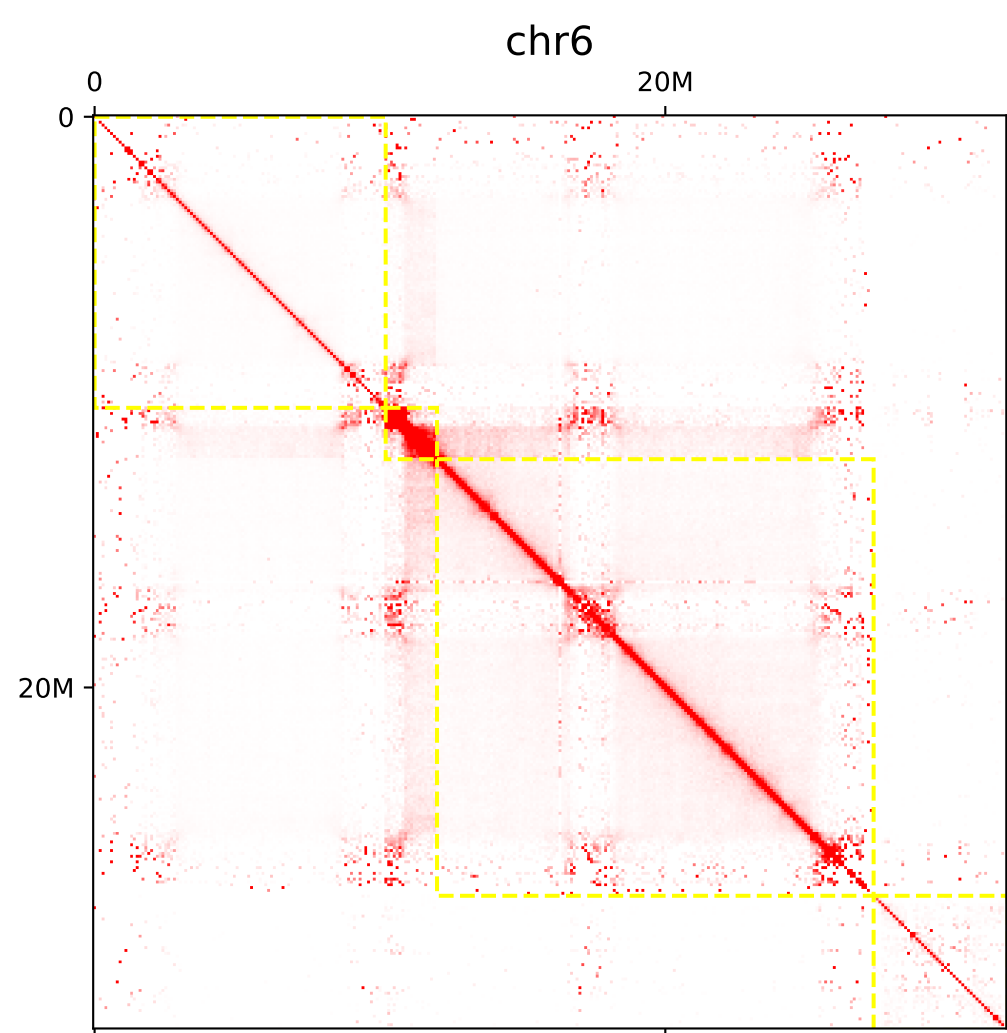

Supplement: Web_Material_uhad111 [file web_material_uhad111.zip › Figure_S5.pdf]

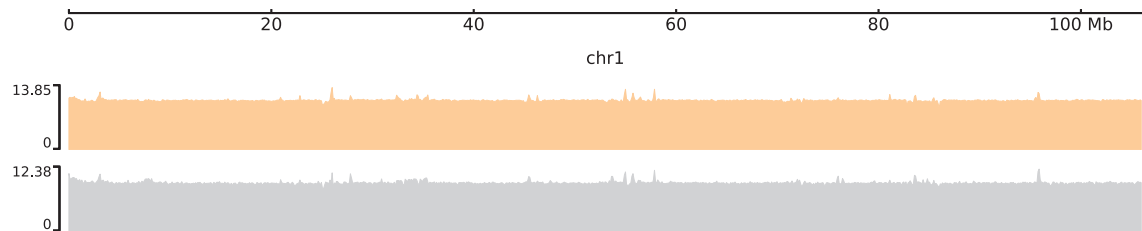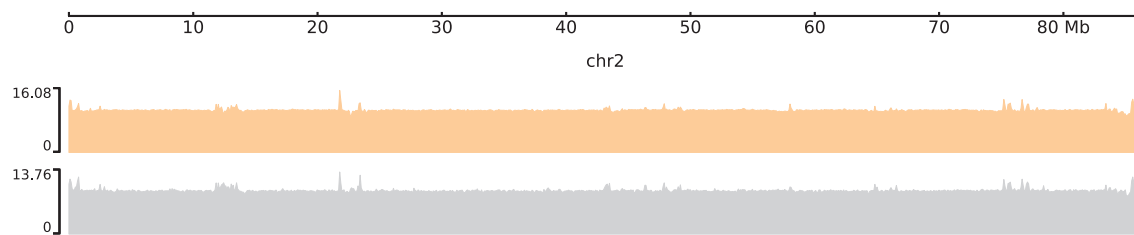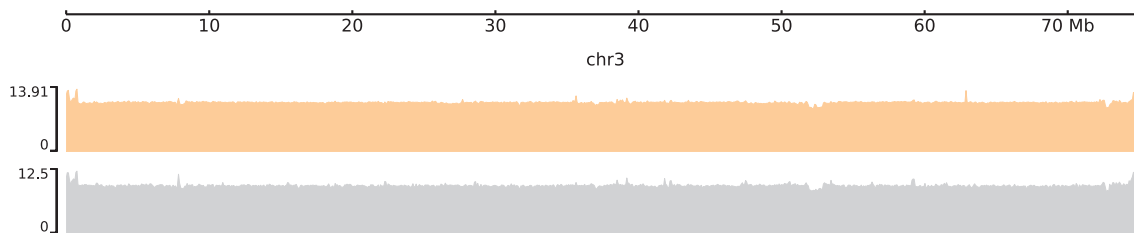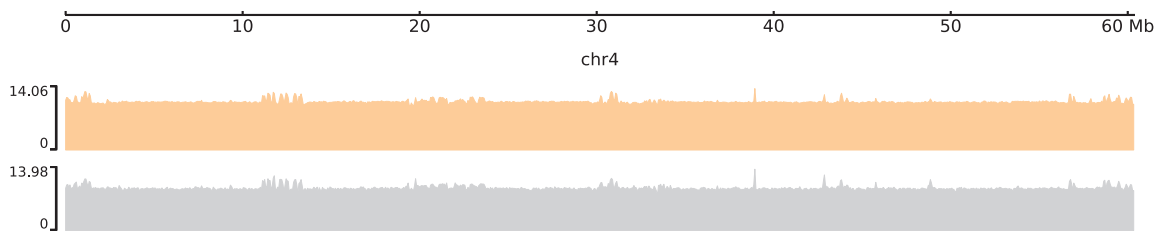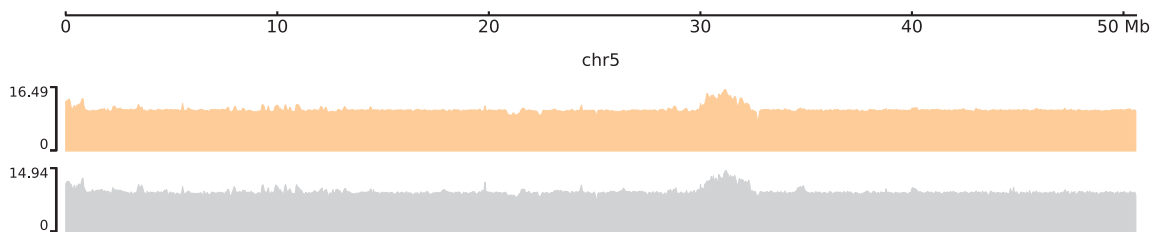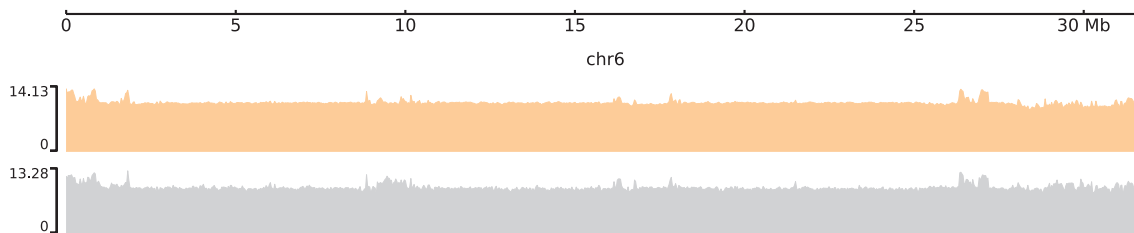

Supplement: Web_Material_uhad111 [file web_material_uhad111.zip › Figure_S6.pdf]

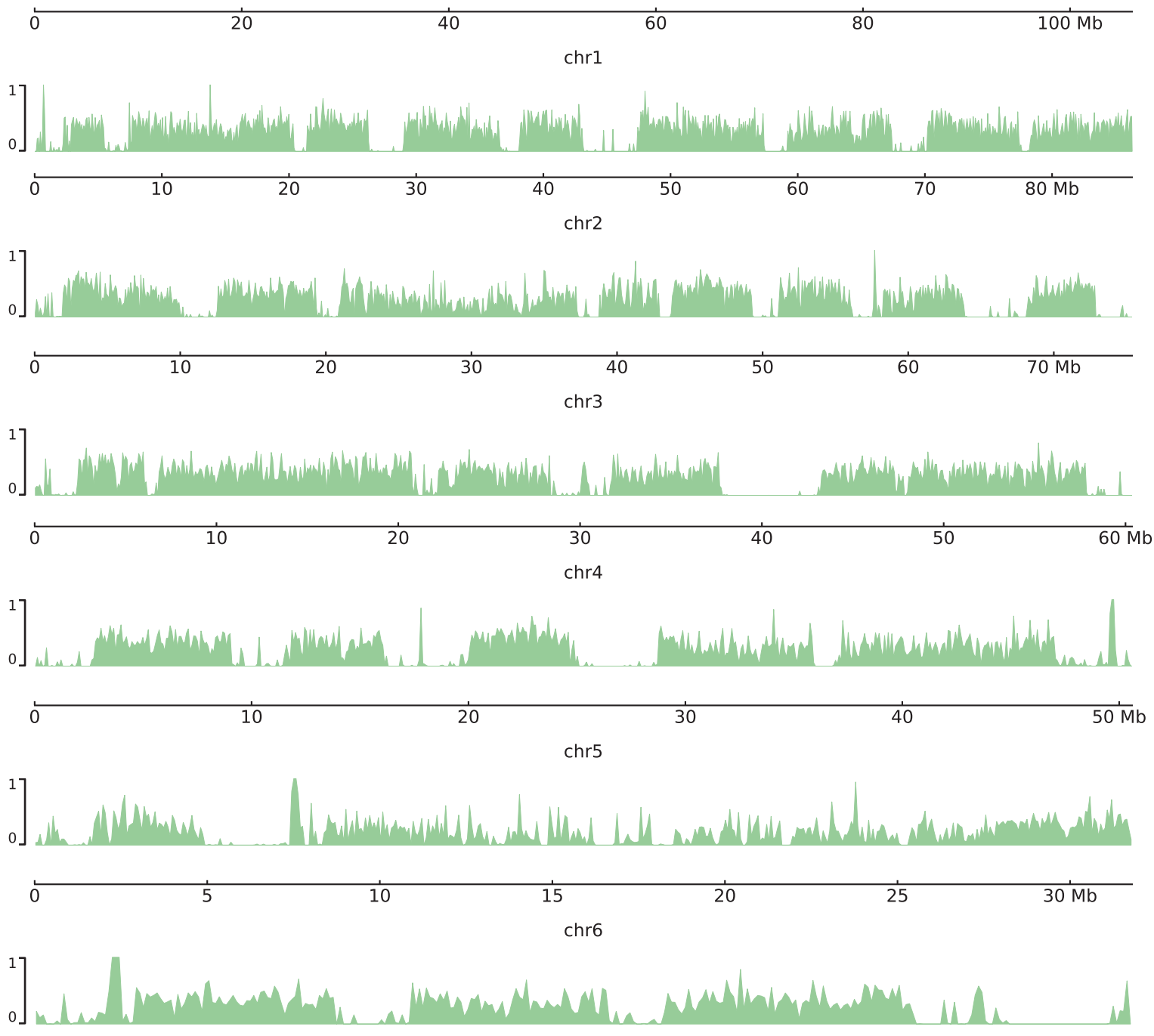

Supplement: Web_Material_uhad111 [file web_material_uhad111.zip › Figure_S7.pdf]

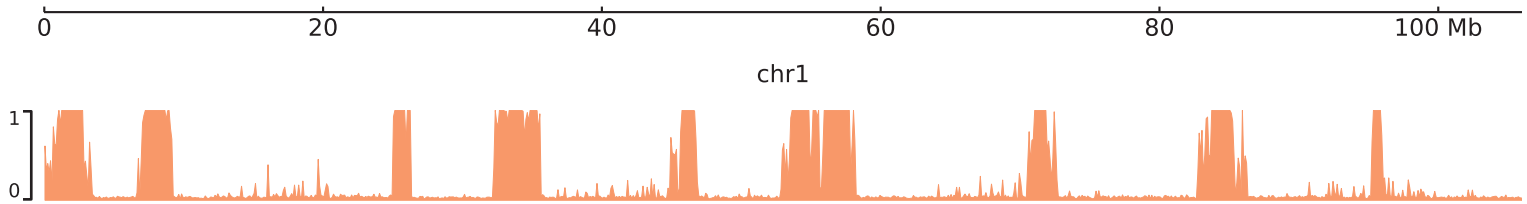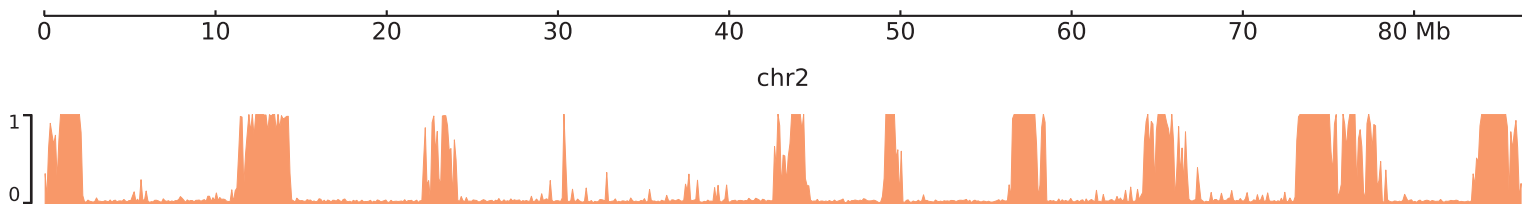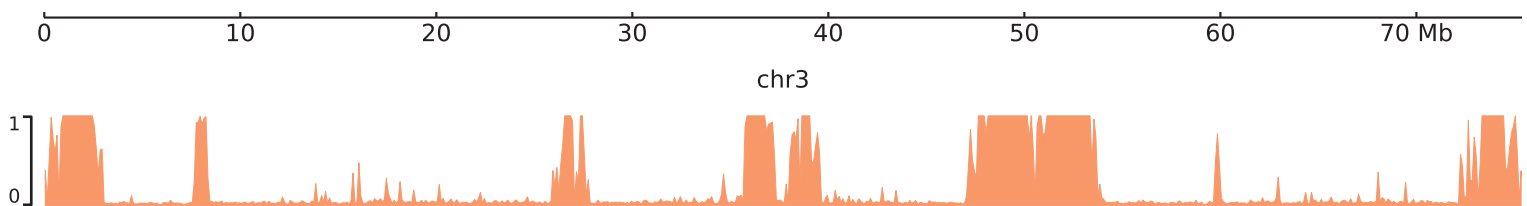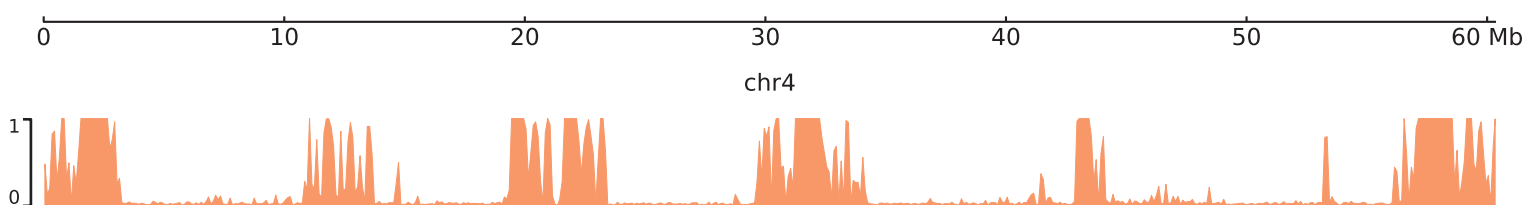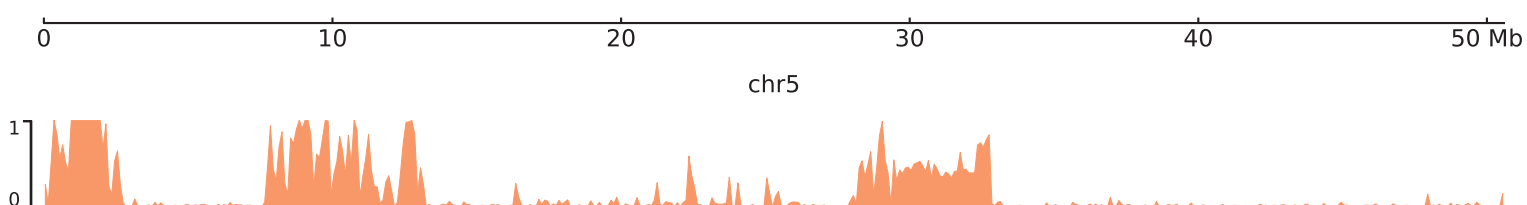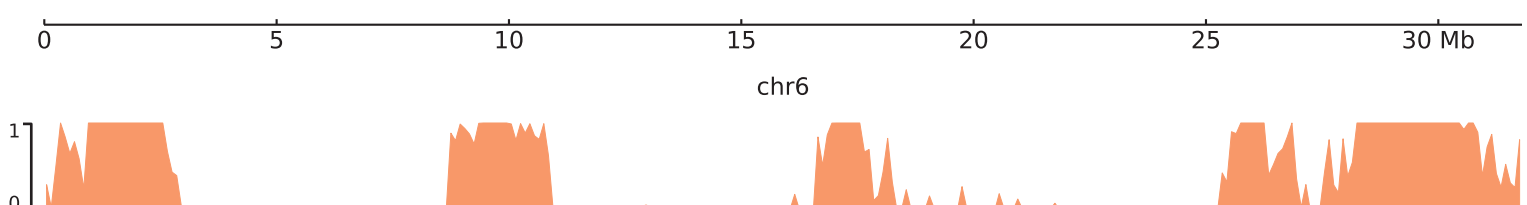

Supplement: Web_Material_uhad111 [file web_material_uhad111.zip › Figure_S8.pdf]

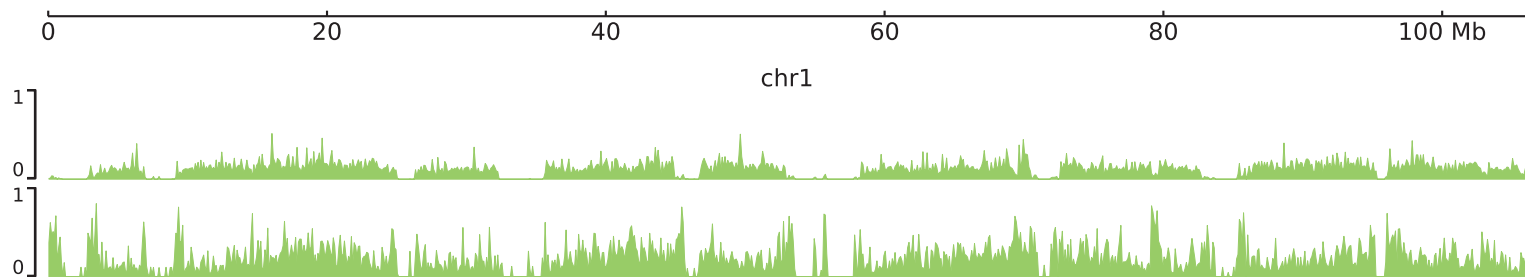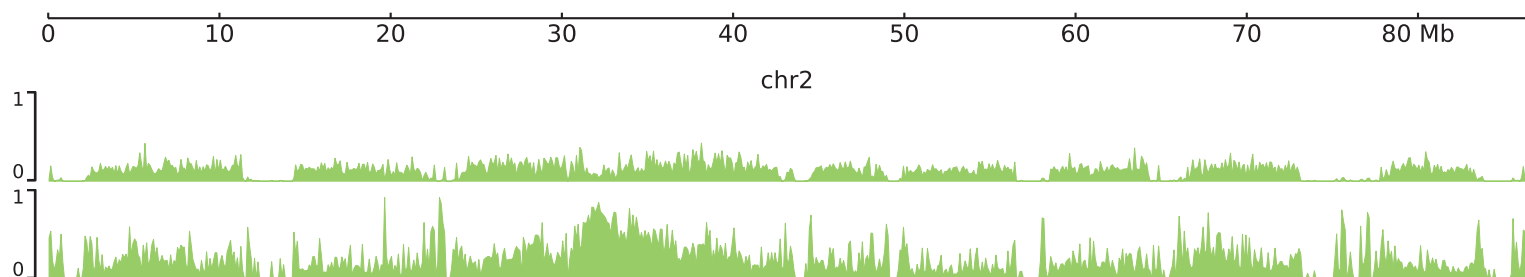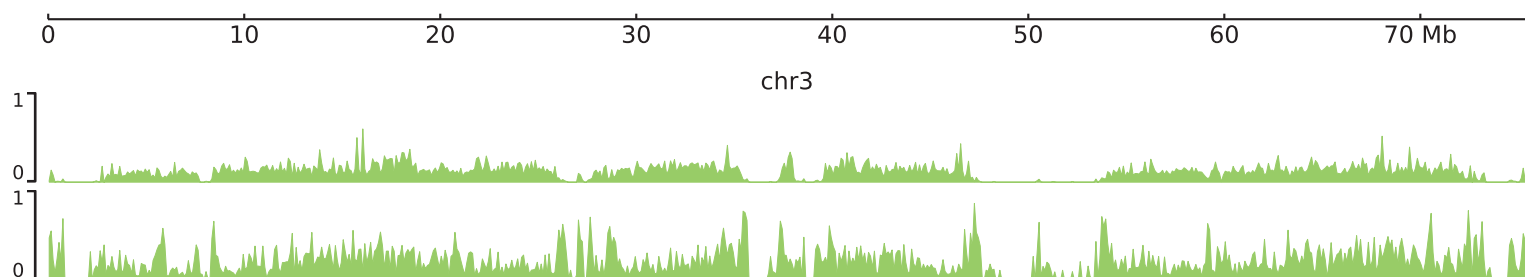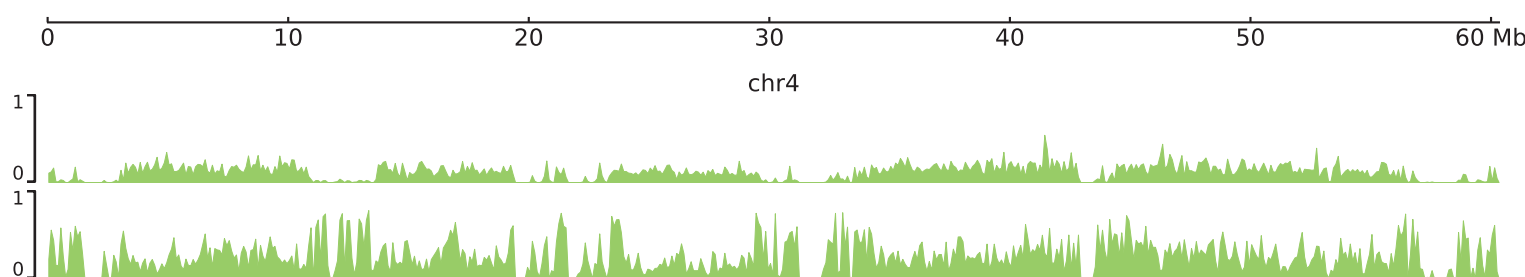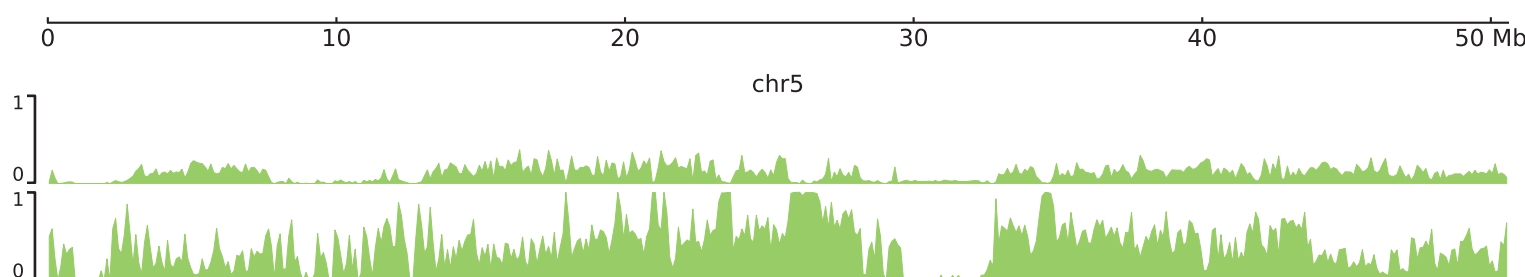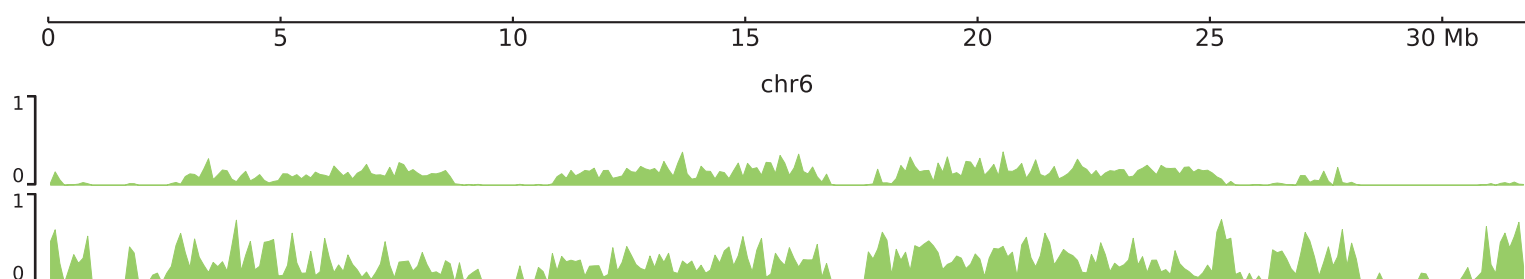

Supplement: Web_Material_uhad111 [file web_material_uhad111.zip › Figure_S9.pdf]

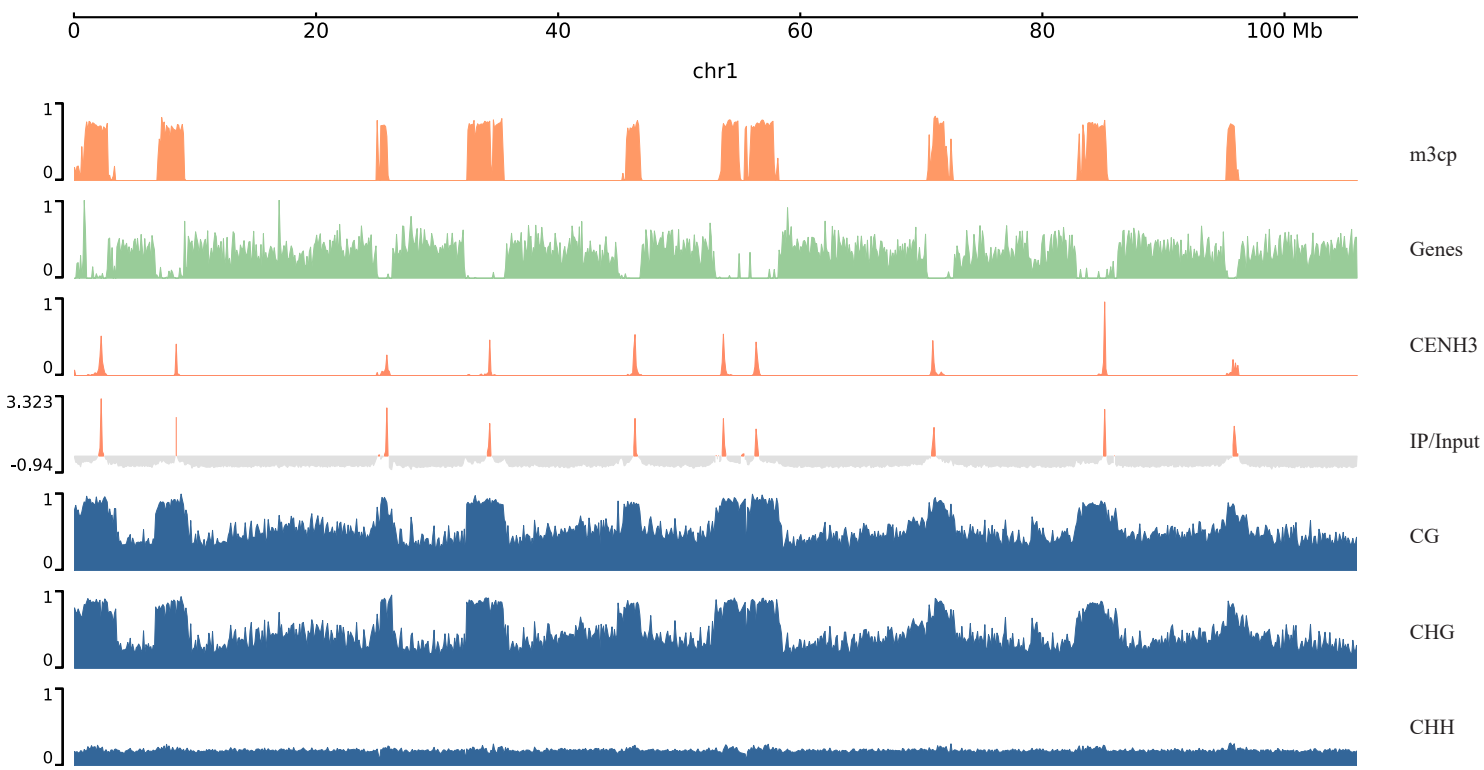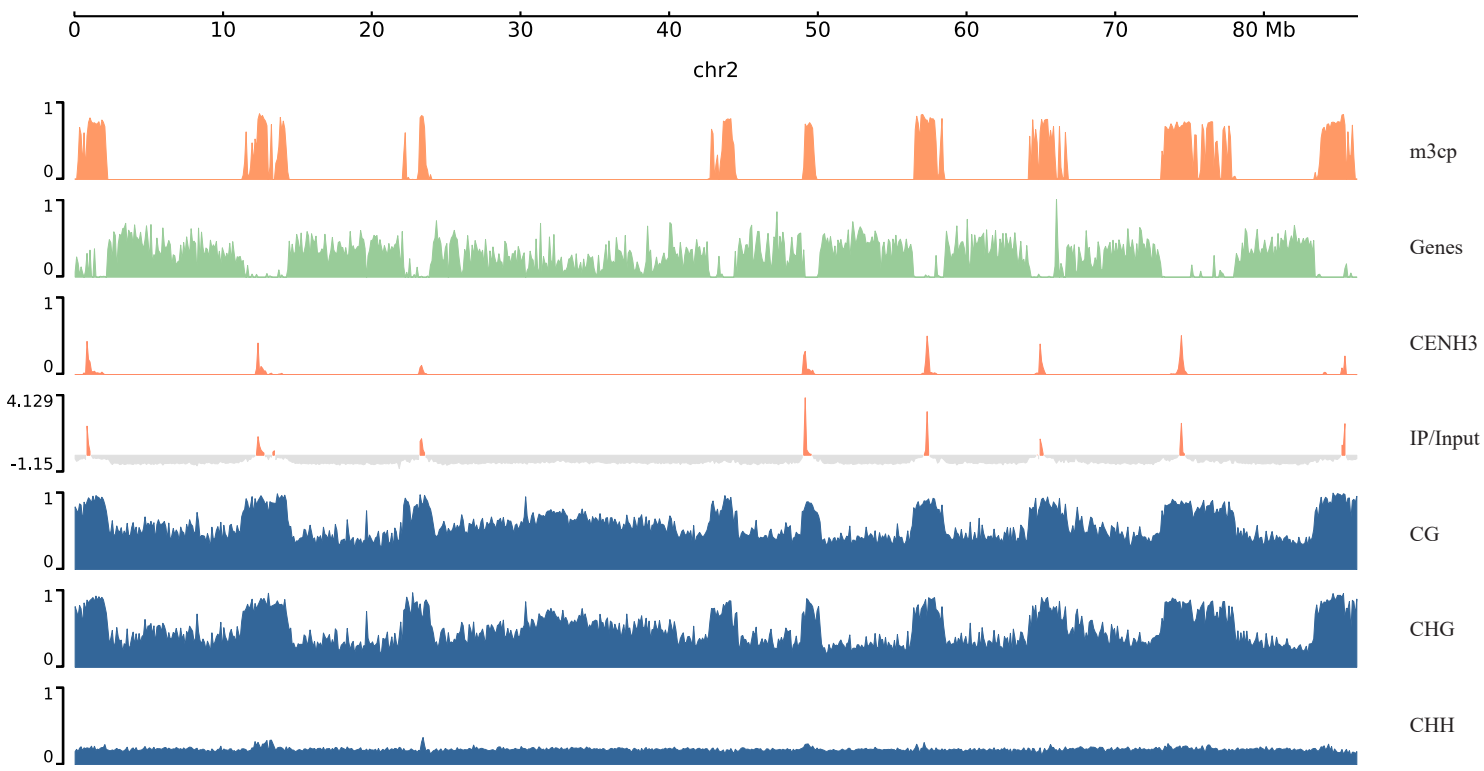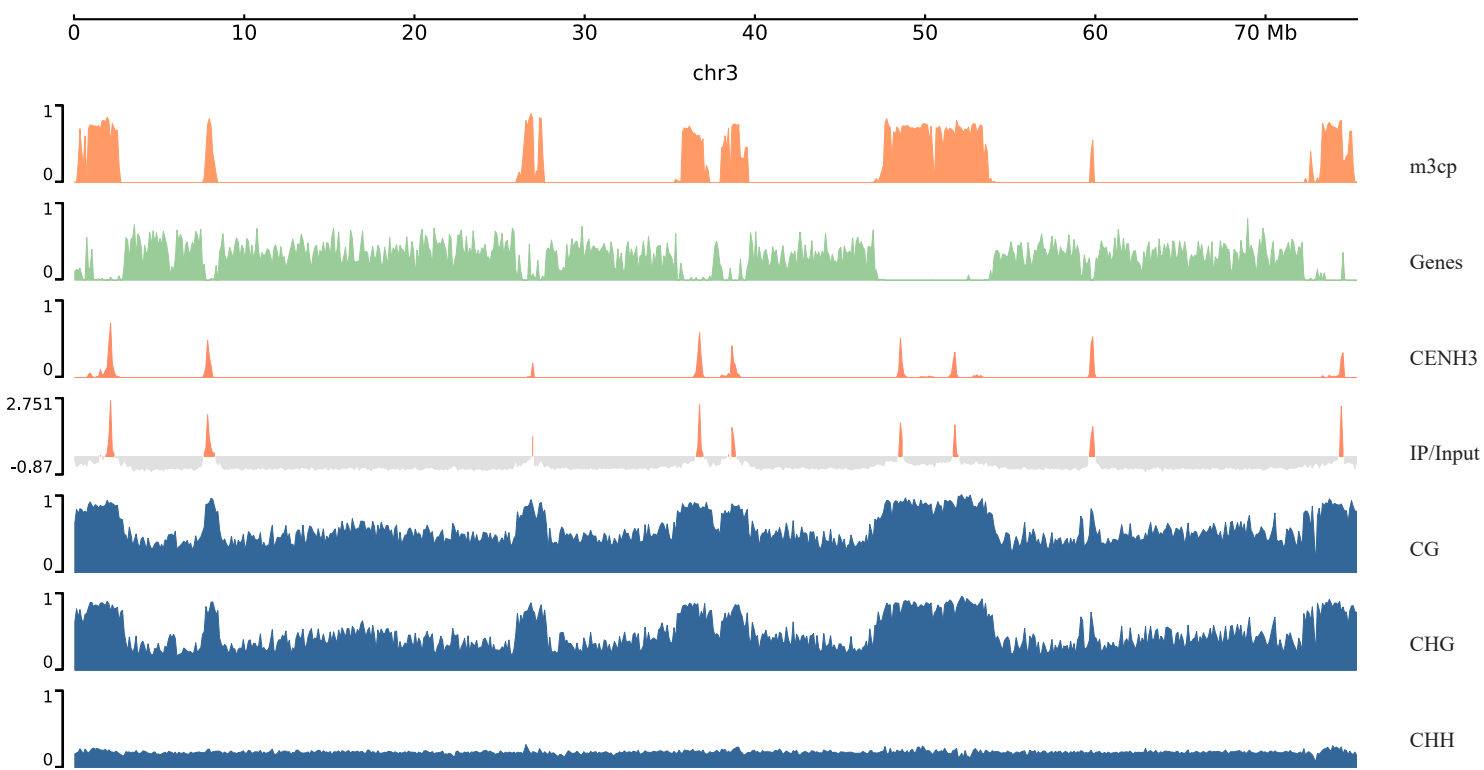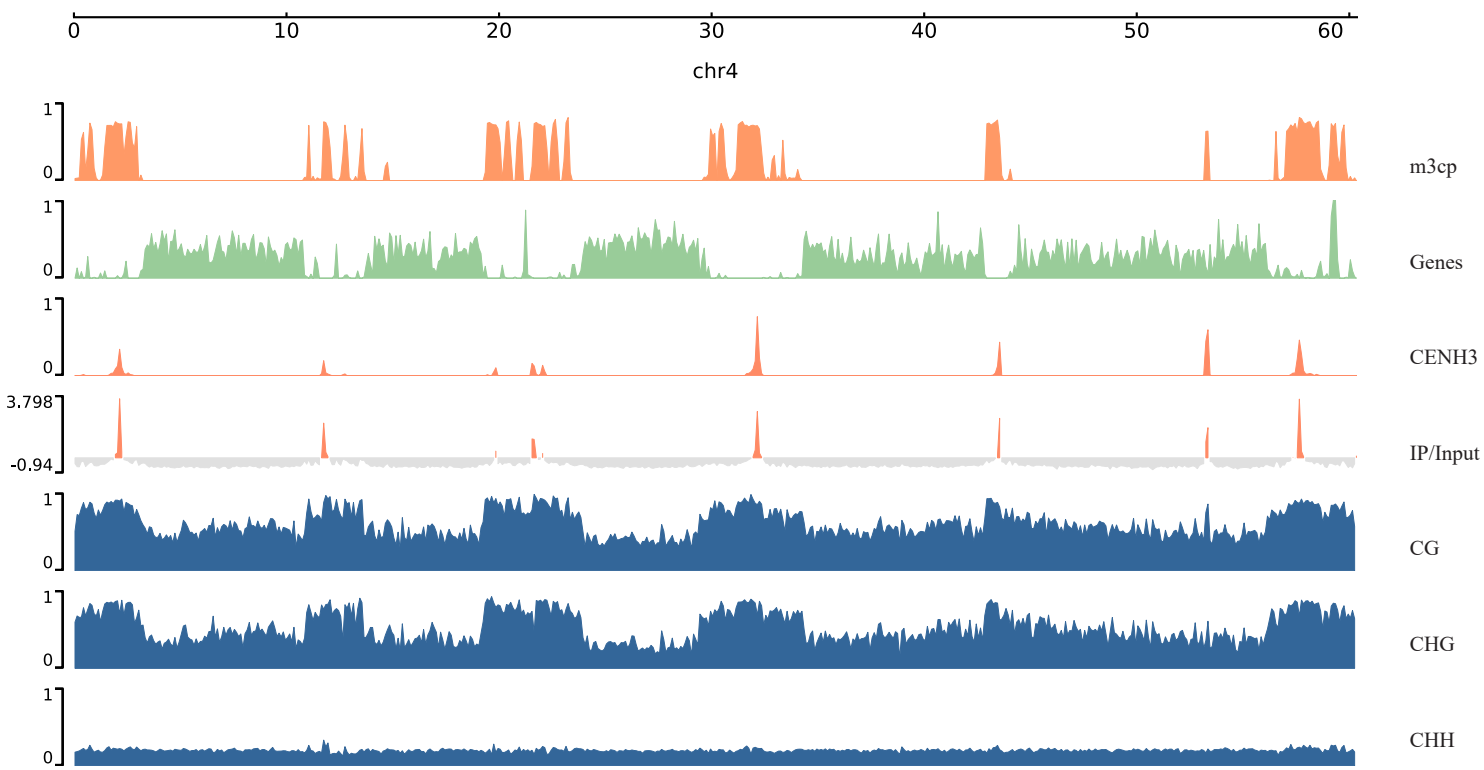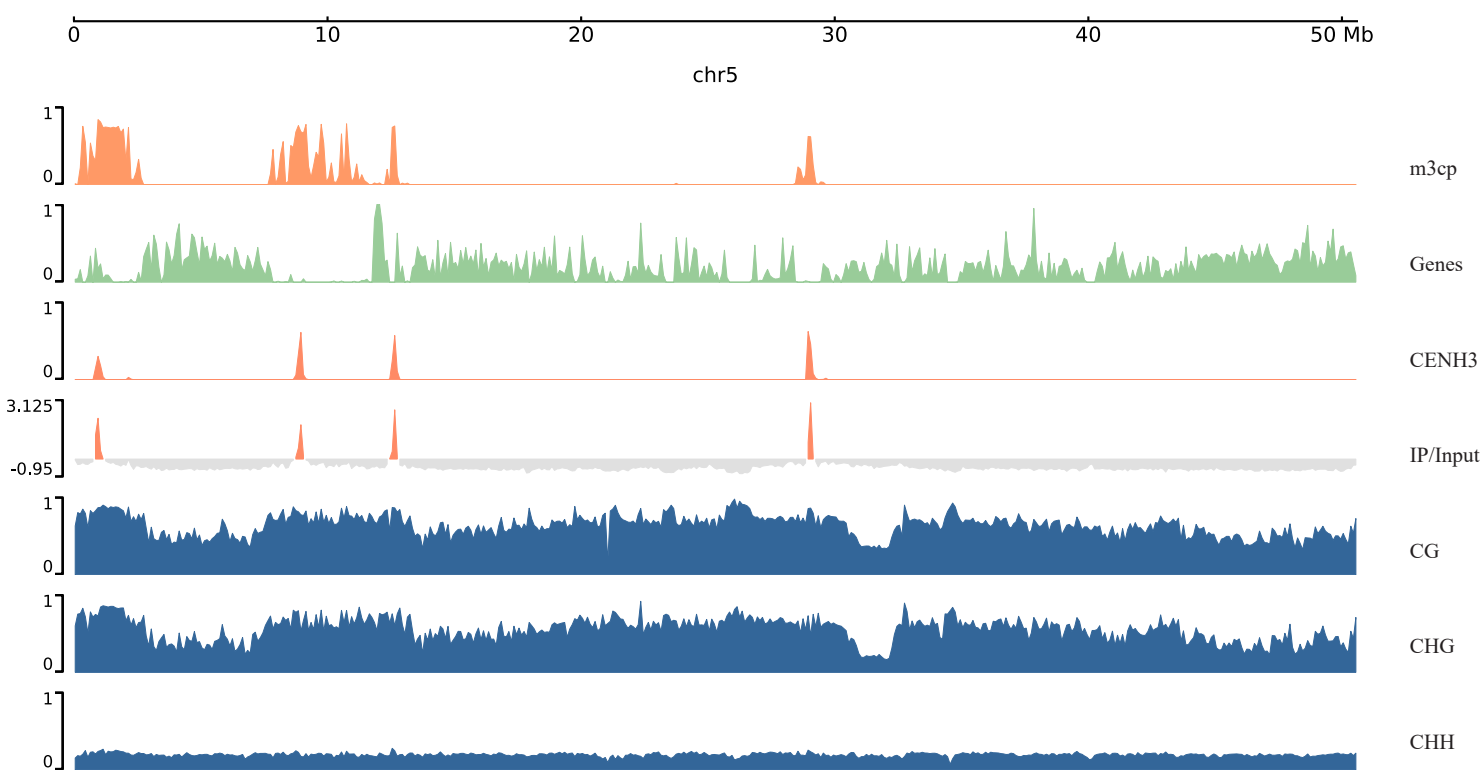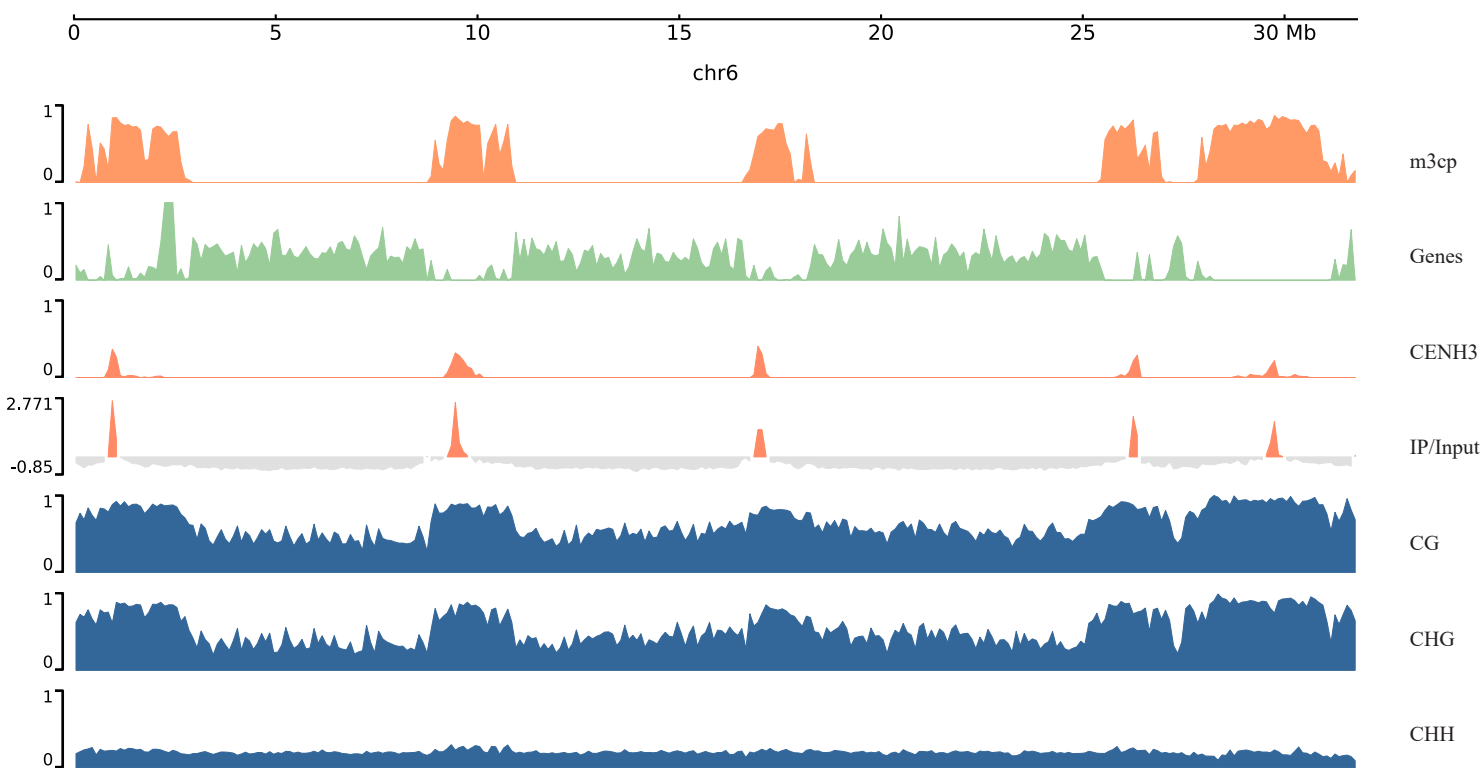

Supplement: Web_Material_uhad111 [file web_material_uhad111.zip › Figure_S10.pdf]

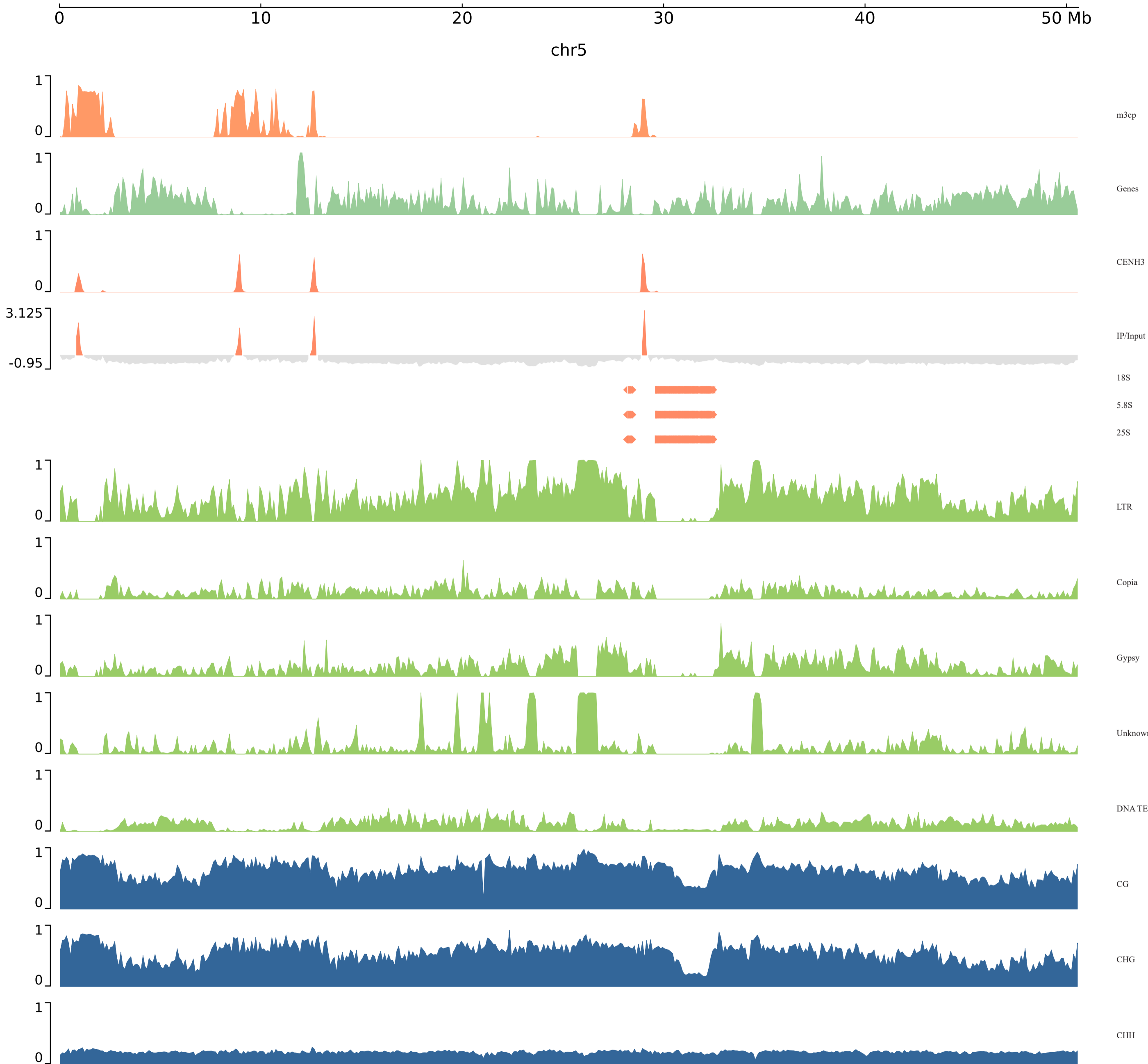

Supplement: Web_Material_uhad111 [file web_material_uhad111.zip › Figure_S11.pdf]

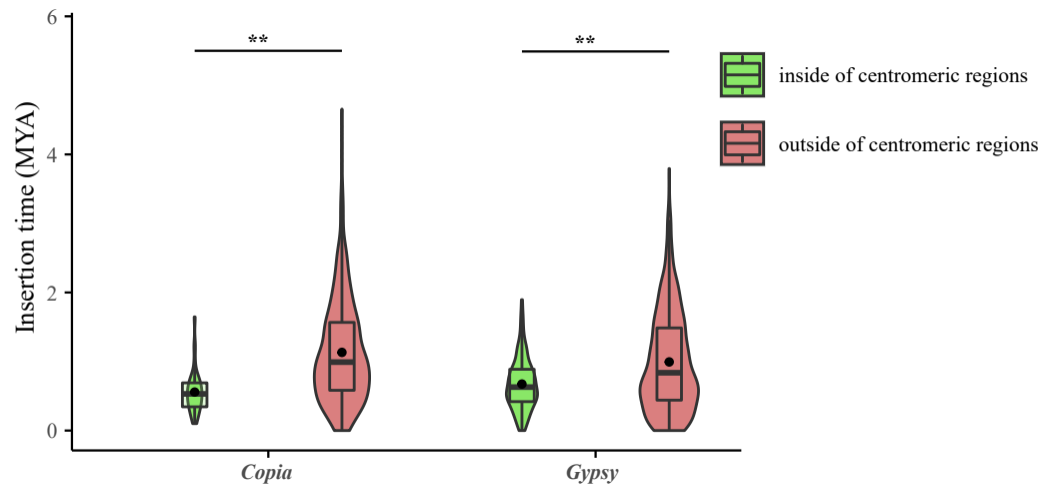

Supplement: Web_Material_uhad111 [file web_material_uhad111.zip › Figure_S12.pdf]
